# hMRAPα, but Not hMRAP2, Enhances hMC4R Constitutive Activity in HEK293 Cells and This Is Not Dependent on hMRAPα Induced Changes in hMC4R Complex N-linked Glycosylation

**DOI:** 10.1371/journal.pone.0140320

**Published:** 2015-10-15

**Authors:** Emma I. Kay, Rikus Botha, Johanna M. Montgomery, Kathleen G. Mountjoy

**Affiliations:** 1 Department of Physiology, University of Auckland, Private Bag 92019, Auckland, 1142, New Zealand; 2 Department of Molecular Medicine and Pathology, University of Auckland, Private Bag 92019, Auckland, 1142, New Zealand; 3 Faculty of Medical and Health Sciences, Centre for Brain Research, University of Auckland, Private Bag 92019, Auckland, 1142, New Zealand; 4 Maurice Wilkins Centre for Molecular Biodiscovery, University of Auckland, Private Bag 92019, Auckland, 1142, New Zealand; Louisiana State University Health Sciences Center, UNITED STATES

## Abstract

MRAP1 but not MRAP2, is essential for melanocortin receptor 2 functional expression. Human MRAP1 splice variant (hMRAPα) and human MRAP2 (hMRAP2) also interact with the other melanocortin receptor subtypes *in vitro*, although the physiological significance of these interactions is unknown. Previously we showed that HA-hMC4R co-expression with hMRAPα, but not hMRAP2, specifically alters HA-hMC4R complex N-linked glycosylation. hMRAPα-FLAG also enhances hMC4R constitutive activity *in vitro*. Here we directly compare hMRAPα and hMRAP2 effects on hMC4R constitutive activity in HEK293 cells. In contrast to hMRAPα, co-expression with hMRAP2 had no effect on HA-hMC4R or untagged hMC4R constitutive coupling to adenylyl cyclase. We used fixed and live cell imaging of HA-hMC4R and hMC4R-eGFP respectively, to further characterise effects of hMRAPα on hMC4R subcellular trafficking. hMRAPα-FLAG co-expression did not alter the partitioning of either HA-hMC4R or hMC4R-eGFP into either the ER or the Golgi apparatus, therefore the hMRAPα effect on hMC4R complex N-linked glycosylation is probably not due to hMC4R retention in the ER. We also observed that unlike HA-hMC4R, hMC4R-eGFP lacks complex glycosylation both in the presence and absence of hMRAPα, although both HA-hMC4R and hMC4R-eGFP exhibited increased constitutive coupling to adenylyl cyclase following co-expression with hMRAPα. We conclude that hMRAPα and not hMRAP2 modulates hMC4R constitutive activity. Furthermore, hMRAPα does not increase hMC4R constitutive activity by altering hMC4R complex N-linked glycosylation. Instead we hypothesise that hMRAPα alters hMC4R conformational states leading to increased hMC4R constitutive activity.

## Introduction

Two melanocortin receptor accessory protein genes known as *MRAP1* and *MRAP2* exist in most vertebrates. In most species, MRAP1 but not MRAP2, is essential for functional expression of the melanocortin receptor-2 (MC2R) and glucocorticoid production [1]. However MRAPα, an hMRAP1 splice variant, and MRAP2 are expressed in a range of tissues with no detectable MC2R expression, but where other MCR subtypes are expressed. Human MRAP2 is predominantly expressed in the brain and adrenal gland, while hMRAPα mRNA expression has been detected in a number of additional tissues [1–3]. It is likely that hMRAPα influences hMCR function in tissues other than the brain and adrenal gland. hMRAPα and hMRAP2 also interact with the other four human melanocortin receptor (hMCR) subtypes when they are co-expressed *in vitro*, but the physiological significance of these interactions is poorly understood [2].

We previously showed that hMRAPα-FLAG co-expression with hMCR subtypes in HEK293 cells has differential effects on the functional expression of all five hMCR subtypes. Notably, hMRAPα-FLAG specifically enhanced hMC4R constitutive activity [3]. Recently rare mutations in hMRAP2 have been shown to underlie the development of severe human obesity, and *in vitro* studies showed that this might be due to MRAP2 effects on MC4R signalling [4]. Sebag *et al*. showed that expression of mouse MRAP2 or the zebrafish homologue MRAP2b with the mouse or zebrafish MC4R respectively, reduced MC4R constitutive activity and increased MC4R sensitivity to α-MSH and coupling to adenylyl cyclase [5]. Given that MC4R constitutive activity is hypothesised to be physiologically significant in human body weight regulation [6, 7], it is important to understand how human MRAP proteins may influence hMC4R constitutive activity. Point mutations in the hMC4R N-terminal domain, second and third intracellular loops and in the second, third, fourth and sixth transmembrane domains, result in either increased or decreased constitutive activity when mutant hMC4R are expressed in HEK293 cells and these mutations are associated with obesity [6, 7]. Furthermore, MC4R neurons in the hypothalamic paraventricular nucleus of MC4R-GFP transgenic mice are tonically regulated by metabolic state and the firing of these neurons to inhibit food intake is partly regulated by MC4R constitutive activity, which supports a physiological role for MC4R constitutive activity [8]. In this study we aimed to directly compare hMRAPα and hMRAP2 effects on hMC4R constitutive coupling to adenylyl cyclase in HEK293 cells. hMRAPα significantly increases hMC4R constitutive coupling to adenylyl cyclase, regardless of the epitope tag on either hMRAPα or hMC4R. In contrast, hMRAP2-FLAG neither increases nor decreases HA-hMC4R constitutive coupling to adenylyl cyclase.

We have previously identified a second differential effect of hMRAP proteins on hMC4R biology. Co-expression with hMRAPα-FLAG altered HA-hMC4R molecular mass and complex glycosylation. By contrast, hMRAP2-FLAG did not alter HA-hMC4R molecular mass, but did appear to increase HA-hMC4R total protein expression [9]. Since hMRAPα-FLAG and HA-hMC4R co-localised intracellularly and not at the cell surface, we hypothesised that hMRAPα interacts with the hMC4R intracellularly to alter N-linked complex glycosylation leading to enhanced hMC4R constitutive activity [3]. In the current study we have used confocal and live cell imaging of tagged hMC4R and hMRAPα recombinant proteins to determine whether hMRAPα influences hMC4R secretory trafficking. Our data show that co-expression with hMRAPα-FLAG does not alter partitioning of either HA-hMC4R into the ER or Golgi apparatus. We also show that in contrast with HA-hMC4R, hMC4R-eGFP lacks complex glycosylation both when expressed alone and when co-expressed with hMRAPα or hMRAPα-FLN.

In summary, co-expression with hMRAPα-FLAG, but not hMRAP2-FLAG, enhances hMC4R constitutive activity *in vitro*. hMRAPα-enhanced hMC4R constitutive activity is not dependent on hMC4R complex glycosylation and we hypothesise that increased hMC4R constitutive activity may result from hMRAPα-induced changes in hMC4R conformational states.

## Materials and Methods

### Materials

α-MSH was purchased from Bachem (Bubendorf, Switzerland). FlAsH-EDT_2_ and ReAsH-EDT_2_ were either purchased from Invitrogen (Carlsbad, CA, USA) or synthesised in the laboratory of Dr Margaret Brimble (University of Auckland). DsRed-ER, encoding a fusion protein consisting of DsRed with the ER targeting sequence of calreticulin at the amino terminal and the ER retrieval sequence KDEL, at the carboxyl terminal, was purchased from Clontech (Mountain View, CA, USA). Human β-1,4-galactosyltransferase 1 (GalTase) with an N-terminal mCherry tag was generated by subcloning GalTase from peGFP-N1 (GalTase-GFP), which has previously been described [10], into mCherry-N1, using XhoI and SalI restriction sites and verified by DNA sequencing.

### Expression Constructs for hMC4R, hMRAPα and hMRAP2

hMC4R with 3x N-terminal HA tags in pcDNA 3.1 was purchased from the Missouri S&T cDNA Resource Centre (Rolla, Missouri, USA). hMC4R in pcDNA Neo has previously been described [11]. To generate hMC4R in eGFP-C1 (hMC4R-eGFP), hMC4R was amplified from hMC4R in pcDNA Neo with the sense primer: (5’ CAA GCG CTG GAA TTC TCC TGC CA 3’) and antisense primer (5’ AAC GCT AGC CGA TAT CTG CTA GAC AAG TCA 3’) using Pfx Platinum polymerase (Invitrogen) and the following conditions: denature (94°C, 3 minutes; 94°C, 30 seconds), anneal (66°C, 40 seconds), elongate (72°C, 1 minute) for 35 cycles. The coding sequence for eGFP was removed from the peGFP-C1 plasmid using Nhe1 and EcoR1 restriction enzymes. The hMC4R PCR product and eGFP coding sequence were then ligated in-frame into Eco47 III and EcoR1 digested eGFP-C1 backbone vector, and the recombinant DNA was verified by DNA sequencing. To generate eGFP-hMC4R, hMC4R in pcDNA Neo was cut with EcoRI and SalI and then ligated in frame into EcoRI and SalI sites in pEGFP-C1 (Clontech). hMC4R-PG was amplified from HA-hMC4R in pcDNA 3.1 with the following primers: (5’ ATC GGA TCC ATG GTG AAC TCC ACC CAC CGT 3’) (sense) and (5’ ATC GAA TTC TTA CTG CAG GGT GCT TCT GGC GCA GCA GCC GGG GCA GCA GCT CTC CAT CTG CTT ATA TCT GCT AGA CAA GTC 3’) (antisense). PCR for hMC4R-PG was performed with iProof High Fidelity DNA polymerase (Bio-Rad Laboratories, Philadelphia, PA, USA) as follows: denature (98°C, 1 minute; 98°C, 5 seconds), anneal (64°C, 30 seconds), elongate (72°C, 30 seconds) for 35 cycles and then one elongation at 72°C, for 7 minutes. hMC4R-PG was subcloned into pcDNA 3.1 using BamHI and EcoRI sites and the recombinant DNA was verified by DNA sequencing (aligned with Genbank accession number NM_005912.2).

hMRAPα-FLAG and hMRAPα in pcDNA 3.1 have previously been described [9]. hMRAPα-FLN and hMRAPα-FLAG-FLN were subcloned into pcDNA 3.1. The tetracysteine tag FLN (FLNCCPGCCMEP) has a strong affinity for ReAsH-EDT_2_ and it was chosen to permit imaging of hMRAPα-FLN together with hMC4R-eGFP or hMC4R-PG [12]. hMRAPα-FLN was amplified from hMRAPα-FLAG in pcDNA 3.1 with the following primers: (5’ ATC GGA TCC ATG GCC AAC GGG ACC AAC) (sense) and (5’ ATC GAA TTC TCA CTC GAG TTA GCG GCC GCC CGG CTC CAT GCA GCA GCC GGG GCA GCA ATT CAA AAA GGA TCC GCT GCT CTC GCT CTG CAA TTG AGA GGT 3’) (antisense). hMRAPα-FLAG-FLN was amplified from hMRAPα-FLAG in pcDNA 3.1 with the following primers: (5’ ATC GGA TCC ATG GCC AAC GGG ACC AAC 3’) (sense) and (5’ ATC GAA TTC TCA CTC GAG TTA GCG GCC GCC CGG CTC CAT GCA GCA GCC GGG GCA GCA ATT CAA AAA GGA TCC GCT GCT CTC CTT GTC ATC GTC GTC CTT 3’) (antisense). PCR for both hMRAPα constructs was performed using iProof High Fidelity DNA polymerase (Bio-Rad Laboratories, Philadelphia, PA, USA) and the following conditions: denature (98°C, 1 minute; 98°C, 5 seconds), anneal (60°C, 30 seconds), elongate (72°C, 10 seconds) for 35 cycles and then one elongation at 72°C, for 7 minutes. hMRAPα-FLN and hMRAPα-FLAG-FLN were subcloned into pcDNA 3.1 using an EcoRV site and the recombinant DNA was verified by sequencing (aligned with Genbank accession number NM_178817.3). hMRAP2 in pcDNA 3.1 has already been described [9]. To subclone hMRAP2-FLAG, human testes total RNA (Clontech; 2 μg) was reverse transcribed to cDNA with Superscript III reverse transcriptase in a total volume of 20 μl and 2 μl of cDNA were used to amplify hMRAP2-FLAG by RT-PCR with the following primers: (5’ ATC GGA TCC ATG TCC GCC CAG AGG TTA AT) (sense) and (5’ ATC GAA TTC TCA CTT GTC ATC GTC GTC CTT GTA GTC ATC CAG GTC TTT GTG TG 3’) (antisense). RT-PCR was performed using iProof High Fidelity DNA polymerase and the following conditions: denature (98°C, 1 minute; 98°C, 5 seconds), anneal (65°C, 30 seconds), elongate (72°C, 10 seconds) for 35 cycles and then one elongation at 72°C, for 7 minutes. hMRAP2-FLAG was subcloned into pcDNA 3.1 using BamHI and EcoRI restriction sites and the recombinant DNA was verified by sequencing. The coding sequence aligned with Genbank accession number NM_138409.2.

### Cell Culture and Transfection

The HEK293 human embryonic kidney cells used in this study were originally sourced from the ATCC and the cells were recently authenticated. For the SNPs tested, they are 100% identical to ATCC clone CRL-12013. HEK293 cells were grown in Dulbecco’s Modified Eagles Medium (DMEM) supplemented with 10% (v/v) newborn calf serum (NCS) and 1% (v/v) penicillin/streptomycin (Invitrogen Corporation, Carlsbad, CA, USA) at 37°C under 5% CO_2_. For transient transfection, HEK293 cells were seeded into 6 well plates, 24 well plates or 35mm fluorodishes (World Precision Instruments Inc, Sarasota, FL, USA). After 48 hours, when cells were at 30–50% confluency, they were transfected with 1.5μl of FuGENE 6 (Roche Applied Science, Indianapolis, IN, USA) for every 0.5μg of plasmid DNA. Transient transfections were incubated for 48 hours at 37°C, 5% CO_2._ Stable cell lines were generated as previously described [3].

For immunocytochemistry, HEK293 cells or HEK293 cells stably transfected with DsRed-ER, GalTase-mCherry or hMC4R-eGFP were seeded onto glass coverslips in 6 well plates prepared as previously described [3]. For live cell imaging, cells were seeded into 35mm fluorodishes (World Precision Instruments Inc, Sarasota, FL, USA) which had been coated with poly-L-lysine for 3 hours at 37°C. Forty-eight hours later when the cells were 30–50% confluent, cells were transiently transfected with HA-hMC4R or hMC4R-eGFP, together with hMRAPα, hMRAPα-FLAG, hMRAP2-FLAG or pcDNA 3.1.

### Immunocytochemistry and Preparation of Cells for Live Cell Imaging

Immunocytochemistry was performed as previously described [9] except that mouse anti-HA.11 monoclonal antibody (Covance Research Products, Princeton, NJ, USA) and rabbit anti-FLAG polyclonal antibody (Sigma Aldrich Ltd, St Louis, MO, USA) were diluted 1:5,000 in PBS. Cells were then washed with PBS for 3x 5 minute intervals prior to incubation with 4 μg/mL goat anti-mouse FITC and 2 μg/mL goat anti-rabbit Alexa 568 or 647 secondary antibodies (Invitrogen, Carlsbad, CA, USA) diluted in PBS + 0.1% BSA, for two hours at room temperature. Following this, cells were washed with PBS for 3x five minute intervals. Nuclei were counter-stained with 600 nM DAPI (Invitrogen, Carlsbad, CA, USA) following staining with secondary antibody. Slides were prepared for confocal microscopy as previously described [3]. For live cell imaging, the culture media was removed 48 hours post-transfection and the cells were washed 1x with warm Hanks Balanced Salt Solution (HBSS) (Invitrogen) + 1 g/L glucose + 25 mM HEPES. Cells were then maintained in HBSS + 1 g/L glucose + 25 mM HEPES for live cell imaging.

### Confocal Microscopy, Live Cell Imaging and Colocalisation Analysis

Fixed cell imaging was performed on an Olympus FV1000 confocal microscope with Olympus Fluoview 1.7 acquisition software. At least three images were acquired for each transfection. For HA-hMC4R transfected cells and HEK293 cells stably expressing hMC4R-eGFP, Z-stack images at 1.0 μm intervals were acquired with a 60x oil immersion lens (numerical aperture = 1.3) and 2x optical zoom. At least three Z-stacks of images were acquired for each transfection. For HEK293 cells transiently-transfected with HA-hMC4R and hMRAP2-FLAG, single slice images were acquired from the approximate centre of each monolayer with a 60x oil immersion lens (numerical aperture = 1.3) and 3x optical zoom. Maximal intensity Z-projections were processed in Image J [13].

Live cell imaging was performed on an Olympus FV1000 confocal microscope housed in a Solent incubation system heated to 37°C. Cells were constantly perfused with HBSS + 1 g/L glucose + 25 mM HEPES at a slow flow rate. Time series images were acquired using a 60x magnification long working distance water immersion objective, 2x optical zoom and Olympus Fluoview 1.7 software. Images were taken from the approximate centre of each monolayer. For each time series, individual frames (512 x 512 pixels) were acquired every 2.2–10 seconds depending on the experiment. At least 3 image series were acquired per transfection.

Images for co-localisation analysis were processed using Image J. First, each cell of interest was manually outlined to generate a region of interest (ROI). Subsequently the Image J plugin, ‘Just Another Colocalisation Plugin’ (JACoP) was used to manually threshold both the red and green channels and then to calculate the M co-efficient for each ROI, which represents the fraction of HA-hMC4R or hMC4R-eGFP fluorescent signal that overlaps with either red or green fluorescent signal [14]. Each coverslip was divided into 9 quadrants and one cell was analysed from each of 3–5 quadrants. Cells that expressed all of the expected proteins for each transfection were selected. Three independent experiments were performed resulting in analysis of a total of 11–15 ROIs per transfection.

### Measurement of Adenylyl Cyclase Activity

HEK293 cells in 24 well plates were transiently transfected with hMC4R, HA-hMC4R, hMC4R-eGFP, eGFP-hMC4R or hMC4R-PG together with hMRAPα, hMRAPα-FLAG, hMRAP2-FLAG, hMRAPα-FLN or the empty vector pcDNA 3.1 as described above. Forty-eight hours post-transfection, cells were stimulated with α-MSH and then adenylyl cyclase activity measured as previously described [3].

### SDS-PAGE and Western Blotting

HEK293 cells grown to 30–50% confluency in 6-well plates were transiently transfected with hMC4R-eGFP plus hMRAPα, hMRAPα-FLN or the empty vector (pcDNA 3.1). Sample preparation for SDS-PAGE and chemiluminescent detection was performed as previously described [9]. To detect hMC4R-eGFP, 15 μg of total cell lysate was separated on 7% bis-acrylamide gels at 4°C and then transferred to PVDF. PVDF membranes were incubated with blocking buffer (TBS + 0.1% Tween 20 + 1% BSA) for 1 hour at room temperature prior to incubation with rabbit anti-GFP polyclonal antibody (Abcam, Cambridge, UK) at 1:10,000 in TBS + 0.1% Tween 20 + 1% BSA, overnight at 4°C. PVDF membranes were then washed 3 times with TBS-T and incubated with ECL horseradish peroxidase conjugated anti-rabbit secondary antibody (GE Healthcare Biosciences, Pittsburgh, PA, USA) at 1:5,000 in TBS + 0.1% Tween 20 + 1% BSA for 1 hour at room temperature.

### Analysis of N-Linked Glycosylation

HEK293 cells grown to 30–50% confluency in 6-well plates were transiently transfected with hMC4R-eGFP plus hMRAPα, hMRAPα-FLN or the empty vector (pcDNA 3.1). Analysis of hMC4R-eGFP N-linked glycosylation was performed as previously described [9] except that 30 μg of total cellular protein was digested with 2 μg of PNGase F in PBS + 1% SDS, for 4 hours at 37°C. For endoglycosidase H digests, 30 μg of total cellular protein was digested with 250 U of recombinant endoglycosidase H (New England Biolabs, Ipswich, MA, USA) for 4 hours at 37°C according to the manufacturer’s instructions. Digested and undigested cell lysates were analysed by SDS-PAGE and western blotting as described above.

### Statistics

Graphpad Prism 5.0 software was used to prepare graphs and to perform statistical analysis. Concentration-response curves were fitted to raw adenylyl cyclase data for statistical comparison of maximum, minimum and EC_50_ values for untagged hMC4R, HA-hMC4R or hMC4R-eGFP coupling to adenylyl cyclase. Adenylyl cyclase activity for untagged hMC4R, HA-hMC4R or hMC4R-eGFP co-expressed with hMRAPα, hMRAPα-FLAG, hMRAP2-FLAG or hMRAPα-FLN was normalised to the minimum and maximum best-fit values for the same hMC4R construct expressed with pcDNA 3.1. Normalised data from at least three independent experiments were pooled and plotted for statistical comparison of maximum, minimum or log EC_50_ values, for untagged or HA-hMC4R coupling to adenylyl cyclase. Normalised data from at least three independent experiments were pooled and plotted for statistical comparison of minimum values for hMC4R-eGFP coupling to adenylyl cyclase. Statistical significance was determined using the non-parametric sum of squares *f* test. M coefficients for HA-hMC4R or hMC4R-eGFP overlap with either DsRed-ER or GalTase-mCherry were statistically compared using one-way ANOVA and Tukey’s multiple comparisons test. Data is expressed as average M for 11–15 ROIs ± s.e.m. A p-value of <0.05 was considered significant.

## Results

### hMRAPα-FLAG, but Not hMRAP2-FLAG, Enhances HA-hMC4R Constitutive Activity in HEK293 Cells

**Fig 1:**
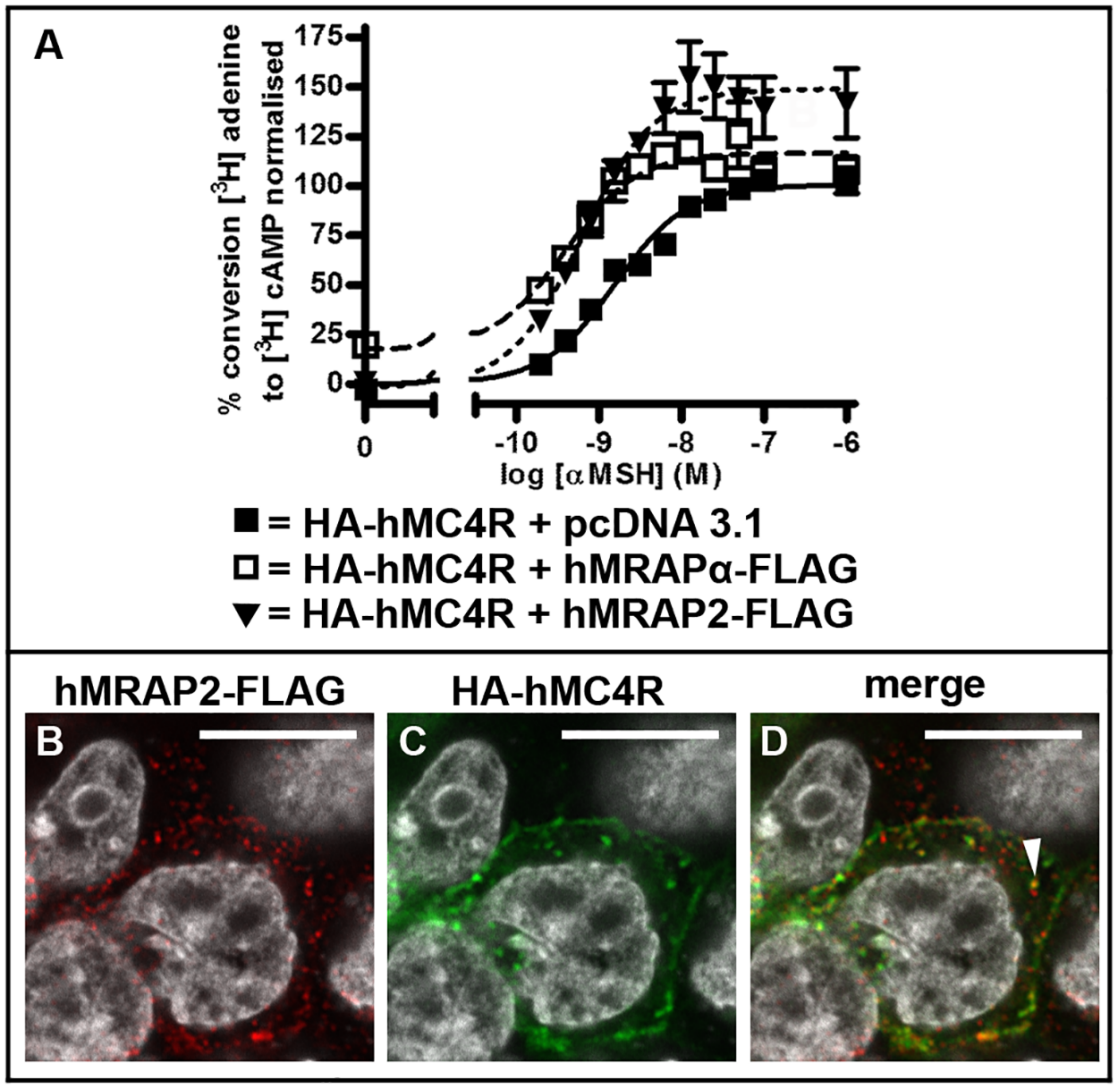
hMRAPα-FLAG, but not hMRAP2-FLAG, enhances HA-hMC4R constitutive activity in HEK293 cells. HA-hMC4R was transiently co-expressed with the empty vector pcDNA 3.1, hMRAPα-FLAG or hMRAP2-FLAG in HEK293 cells (A) and HA-hMC4R coupling to adenylyl cyclase was stimulated with increasing concentrations of α-MSH for 1 hour and adenylyl cyclase activity measured. Normalised data from 3 independent experiments performed in duplicate were pooled and plotted as mean ± s.e.m. Best-fit values for baseline and maximum responses and EC_50_ for these curves are shown in Table 1. To determine whether hMRAP2-FLAG is co-expressed with HA-hMC4R, HA-hMC4R and hMRAP2-FLAG were transiently transfected into HEK293 cells and confocal microscopy was performed (B-D). Representative single slices through the approximate centre of each cell are shown. HEK293 cells were counterstained with Hoechst or DAPI to determine signal orientation. The white arrow indicates signal overlap between HA-hMC4R and hMRAP2-FLAG. Scale bars = 10μm.

We determined that hMRAPα-FLAG co-transfection with HA-hMC4R increased HA-hMC4R constitutive coupling to adenylyl cyclase compared to baseline coupling of HA-hMC4R co-expressed with pcDNA 3.1 (~17% increase, p = 0.02) (Fig 1A, Table 1). This is consistent with the enhanced untagged hMC4R constitutive activity induced by hMRAPα-FLAG that we previously observed [3]. hMRAP2-FLAG co-transfection with HA-hMC4R did not increase nor decrease HA-hMC4R baseline coupling to adenylyl cyclase (Fig 1A, Table 1). Untagged hMRAP2 also did not appear to alter hMC4R baseline coupling to adenylyl cyclase (S1 Fig and Table 1). Co-transfection of HA-hMC4R with hMRAPα-FLAG or hMRAP2-FLAG increased α-MSH stimulated HA-hMC4R maximal coupling to adenylyl cyclase compared to co-transfection of HA-hMC4R with pcDNA 3.1 (Fig 1A, Table 1). The EC_50_ values were significantly lower for HA-hMC4R expressed with hMRAPα-FLAG or with hMRAP2-FLAG compared to that for HA-hMC4R expressed with pcDNA 3.1 (Table 1). We determined that these effects on maximum responsiveness and α-MSH sensitivity are peculiar to the combination of a FLAG tag on the accessory protein and an HA tag on hMC4R. Co-expression with either untagged hMRAPα or untagged hMRAP2 or with untagged hMC4R did not significantly alter hMC4R responsiveness or α-MSH sensitivity [3].

**Table 1:** Comparison of values for HA-hMC4R and hMC4R coupling to adenylyl cyclase in HEK293 cells shown in Fig 1 and S1 Fig For HA-hMC4R + pcDNA 3.1, hMRAPα-FLAG or hMRAP2-FLAG, normalised adenylyl cyclase data are shown as mean ± s.e.m for baseline and maximum responses and EC_50_ values are shown with 95% confidence intervals in brackets. For hMC4R + pcDNA 3.1 or hMRAP2, normalised adenylyl cyclase data are shown as mean ± s.e.m for baseline values only. Statistically significant differences for HA-hMC4R or HA-hMC4R + pcDNA 3.1 were * p < 0.05; *** p < 0.009; **** p < 0.0001. For direct comparison of hMC4R-eGFP and hMC4R coupling to adenylyl cyclase, mean values for raw data are shown ± s.e.m for baseline and maximum responses and EC_50_ values are shown with 95% confidence intervals in brackets.

Transfection	Baseline	Maximum	EC_50_ (nM)
**HA-hMC4R + pcDNA 3.1**	0.224 ± 3.064	100.4 ± 2.008	1.560 (1.186, 2.052)
**HA-hMC4R + hMRAPα-FLAG**	17.65 ± 7.065*	116.6 ± 3.062**	0.388 (0.235, 0.641)***
**HA-hMC4R + hMRAP2-FLAG**	-1.182 ± 9.402	148.9 ± 4.527***	0.619 (0.388, 0.988)**
**hMC4R + pcDNA 3.1**	-0.473 ± 4.720		
**hMC4R + hMRAP2**	0.321 ± 5.008		

### hMRAP2-FLAG Is Expressed in Intracellular Vesicles in HEK293 Cells which Also Express HA-hMC4R

Since hMRAP2-FLAG did not enhance HA-hMC4R baseline coupling to adenylyl cyclase, we sought to determine whether hMRAP2-FLAG is expressed in HEK293 cells which co-express HA-hMC4R. We transiently co-transfected HEK293 cells with HA-hMC4R and hMRAP2-FLAG and performed confocal microscopy. Signal for hMRAP2-FLAG (Fig 1B) was observed in cells also expressing HA-hMC4R (Fig 1C), therefore the absence of an effect of hMRAP2-FLAG on HA-hMC4R constitutive activity is not due to a lack of hMRAP2-FLAG expression in HEK293 cells. Both HA-hMC4R and hMRAP2-FLAG were localised in intracellular vesicles in HEK293 cells, which were occasionally observed to overlap (Fig 1D). The degree of HA-hMC4R signal overlap with hMRAP2-FLAG appeared to be less obvious than we had previously observed for HA-hMC4R and hMRAPα-FLAG [3].

### HA-hMC4R Distribution in the ER and Golgi Apparatus Is Not Affected by Co-Expression with hMRAPα-FLAG

Co-expression with hMRAPα-FLAG, but not with hMRAP2-FLAG, increases HA-hMC4R constitutive activity and alters HA-hMC4R molecular mass and complex N-linked glycosylation [3, 9]. Core N-linked glycosylation is added to proteins in the endoplasmic reticulum (ER), while complex N-linked glycans are added during transit through the Golgi apparatus. hMRAPα-FLAG specific effects on HA-hMC4R function may be due to changes in MC4R trafficking through the ER and Golgi apparatus. To test this hypothesis, we performed confocal microscopy on HEK293 cells transiently transfected with HA-hMC4R and hMRAPα-FLAG and stably expressing either DsRed-ER, a marker of the ER, or GalTase-mCherry, a marker of the Golgi apparatus [10, 15].

**Fig 2:**
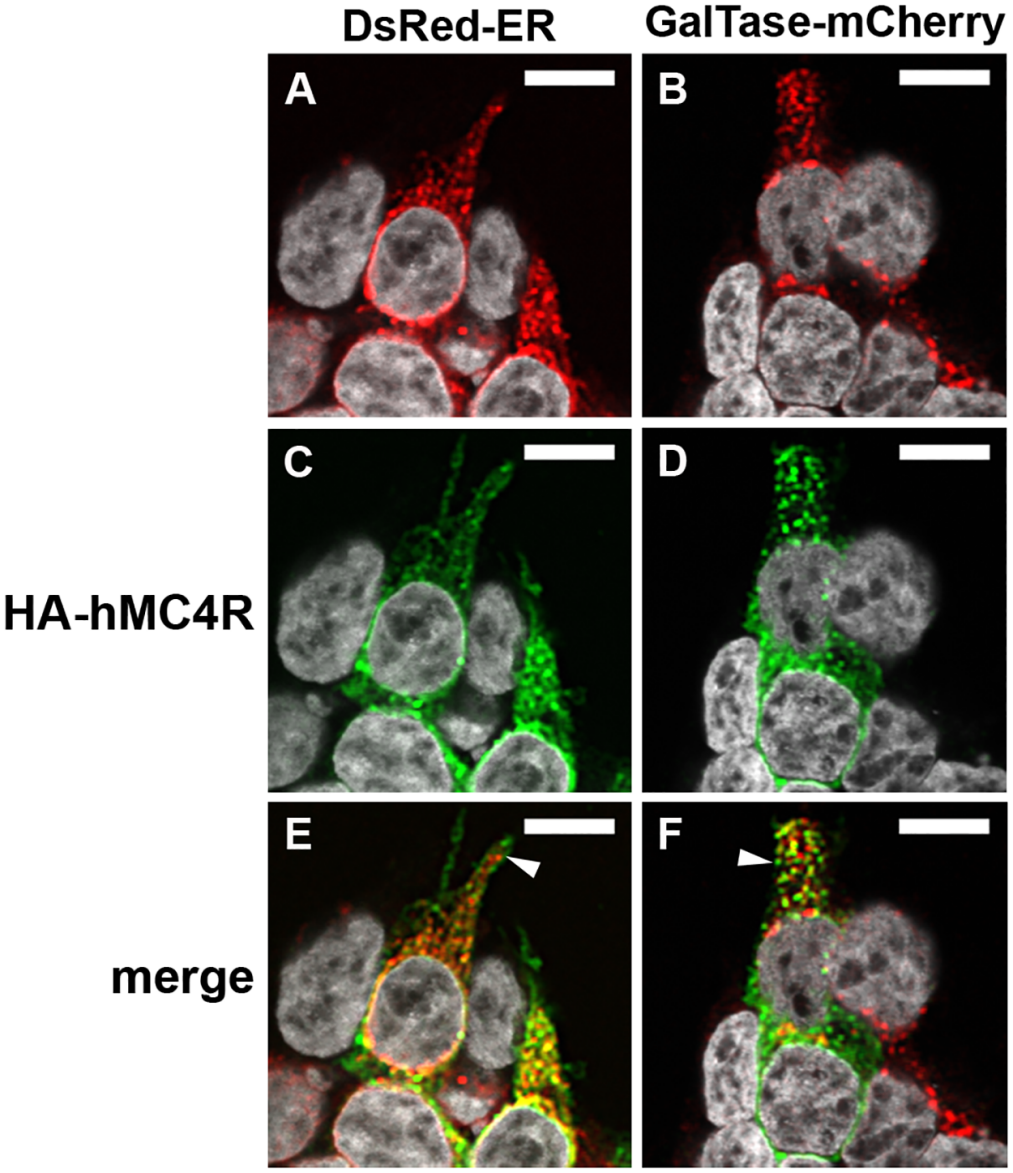
HA-hMC4R co-localised with markers of the ER and Golgi apparatus in HEK293 cells. HA-hMC4R was transiently transfected into HEK293 cells stably expressing DsRed-ER, a marker of the ER (A, C, E) or GalTase-mCherry, a marker of the Golgi apparatus (B, D, F). Z-stacks for HA-hMC4R with each marker were acquired using confocal microscopy and representative single slices through the approximate centre of each cell are shown. HEK293 cells were counterstained with Hoechst or DAPI to determine signal orientation. The white arrows indicate HA-hMC4R signal in close proximity to the cell membrane. Co-localisation analysis for the data represented in this figure is shown in S2A Fig Co-localisation of HA-hMC4R and DsRed-ER or GalTase-mCherry is observed as yellow spots. Scale bars = 10μm.

**Fig 3:**
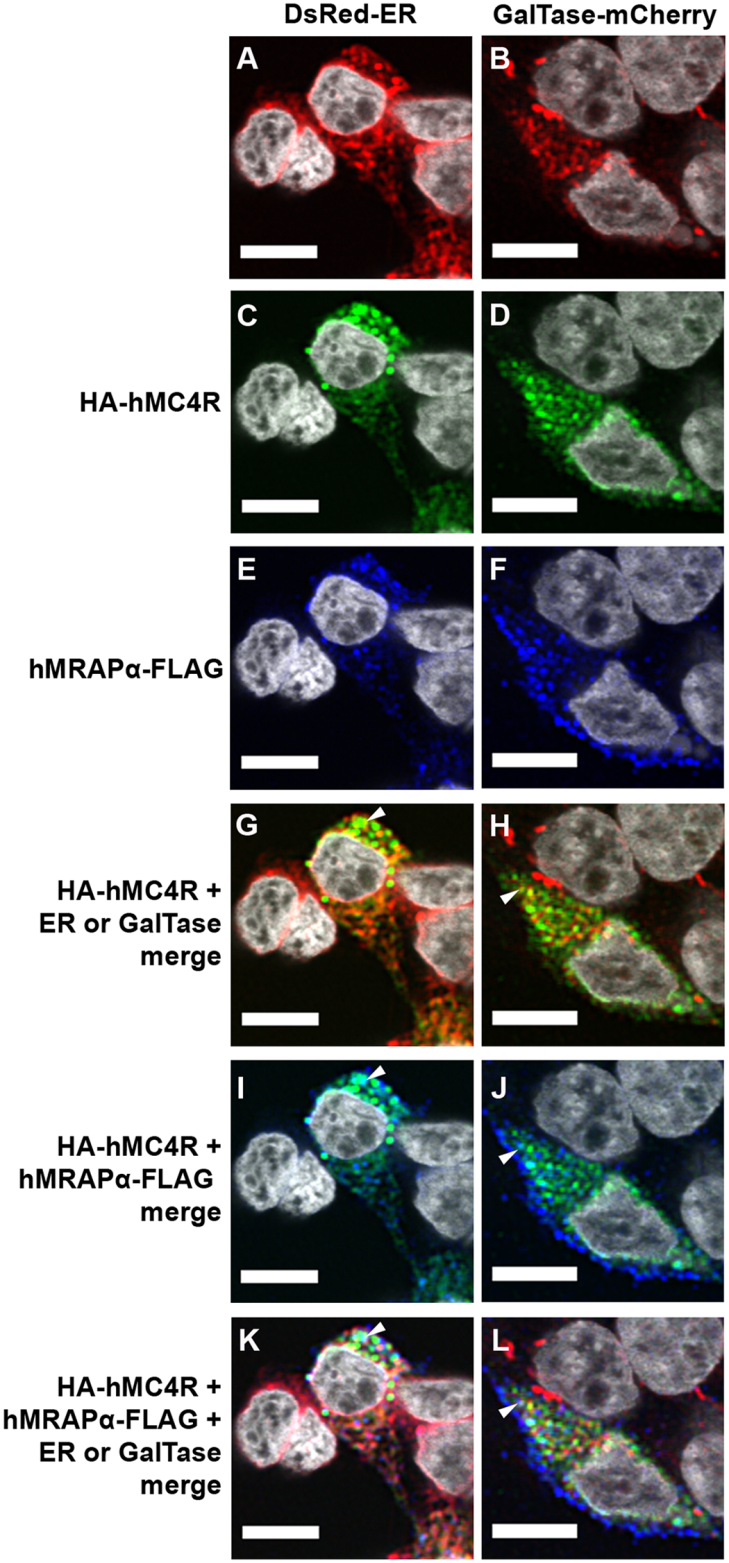
HA-hMC4R and hMRAPα-FLAG co-localised together with markers of the Golgi apparatus and the ER in HEK293 cells. HA-hMC4R and hMRAPα-FLAG were transiently transfected into HEK293 cells stably expressing DsRed-ER, a marker of the endoplasmic reticulum (A, C, E, G, I, K) or GalTase-mCherry, a marker of the Golgi apparatus (B, D, F, H, J, L). Co-localisation of HA-hMC4R and DsRed-ER or GalTase-mCherry is observed as yellow spots and marked by a white arrow in panels G and H. HA-hMC4R and hMRAPα-FLAG co-localisation is observed as aqua coloured spots and marked by a white arrow in panels I and J. Co-localisation of HA-hMC4R and hMRAPα-FLAG and DsRed-ER or GalTase-mCherry is observed as whitish spots and marked by a white arrow in panels K and L. Co-localisation for the data represented by panels G and H in this figure is shown in S2A Fig Scale bars = 10μm.

We examined the subcellular localisation of HA-hMC4R expressed alone (Fig 2) or together with hMRAPα-FLAG (Fig 3) in DsRed-ER or GalTase-mCherry stable cells. In DsRed-ER stable cells, DsRed-ER was expressed at variable levels in predominantly perinuclear locations (Fig 2A). In GalTase-mCherry stable cells, GalTase-mCherry had a punctate expression throughout the cytoplasm (Fig 2B). When HA-hMC4R was expressed alone or with hMRAPα-Flag in DsRed-ER stable cells, 48.3 ± 4% or 50.7 ± 5% (respectively) of the HA-hMC4R puncta overlapped with DsRed-ER (Figs 2E and 3G and S2A Fig). The HA-hMC4R signal that co-localised with DsRed-ER was observed as clusters in the perinuclear cytoplasm (Fig 2C). When HA-hMC4R was expressed alone or with hMRAPα-Flag in GalTase-mCherry stable cells (28.7 ± 4% or 30.1 ± 5% (respectively) of the HA-hMC4R puncta overlapped with GalTase-mCherry (Figs 2F and 3H and S2A Fig). HA-hMC4R signal that did not co-localise with DsRed-ER or GalTase-mCherry was often observed in close proximity to the cell membrane (Fig 2E and 2F). When HA-hMC4R was expressed with hMRAPα-FLAG in DsRed-ER (Fig 3I) or GalTase-mCherry (Fig 3J) stable cells, HA-hMC4R and hMRAPα-FLAG were observed to co-localise similarly to what we have previously reported [3].

A significantly larger fraction of HA-hMC4R signal overlapped with DsRed-ER than with GalTase-mCherry both when HA-hMC4R was expressed alone, and co-expressed with hMRAPα-FLAG (p<0.05) (Figs 2 and 3 and S2A Fig). hMRAPα-FLAG co-expression with HA-hMC4R therefore had no significant effect on distribution of HA-hMC4R partition into either the ER or the Golgi apparatus (S2A Fig).

### Development of a Strategy for Live Cell Imaging of MC4R and hMRAPα

While there appeared to be no difference in MC4R partitioning between the ER and Golgi apparatus following co-expression with hMRAPα-FLAG, we hypothesised that this was because our confocal microscopy study was a ‘snapshot in time’, and therefore not a true reflection of the effects of hMRAPα on hMC4R trafficking. Thus we sought to develop tools to study the dynamics of hMC4R secretory trafficking in the presence and absence of hMRAPα overexpression. As neither hMRAPα-FLAG nor HA-hMC4R were suitable for this purpose, we needed to develop additional constructs for MC4R and MRAPα live cell imaging. Ideally, we aimed to monitor hMRAPα and hMC4R simultaneously by live cell imaging and use tags that had negligible impact on the function of these proteins.

**Fig 4:**
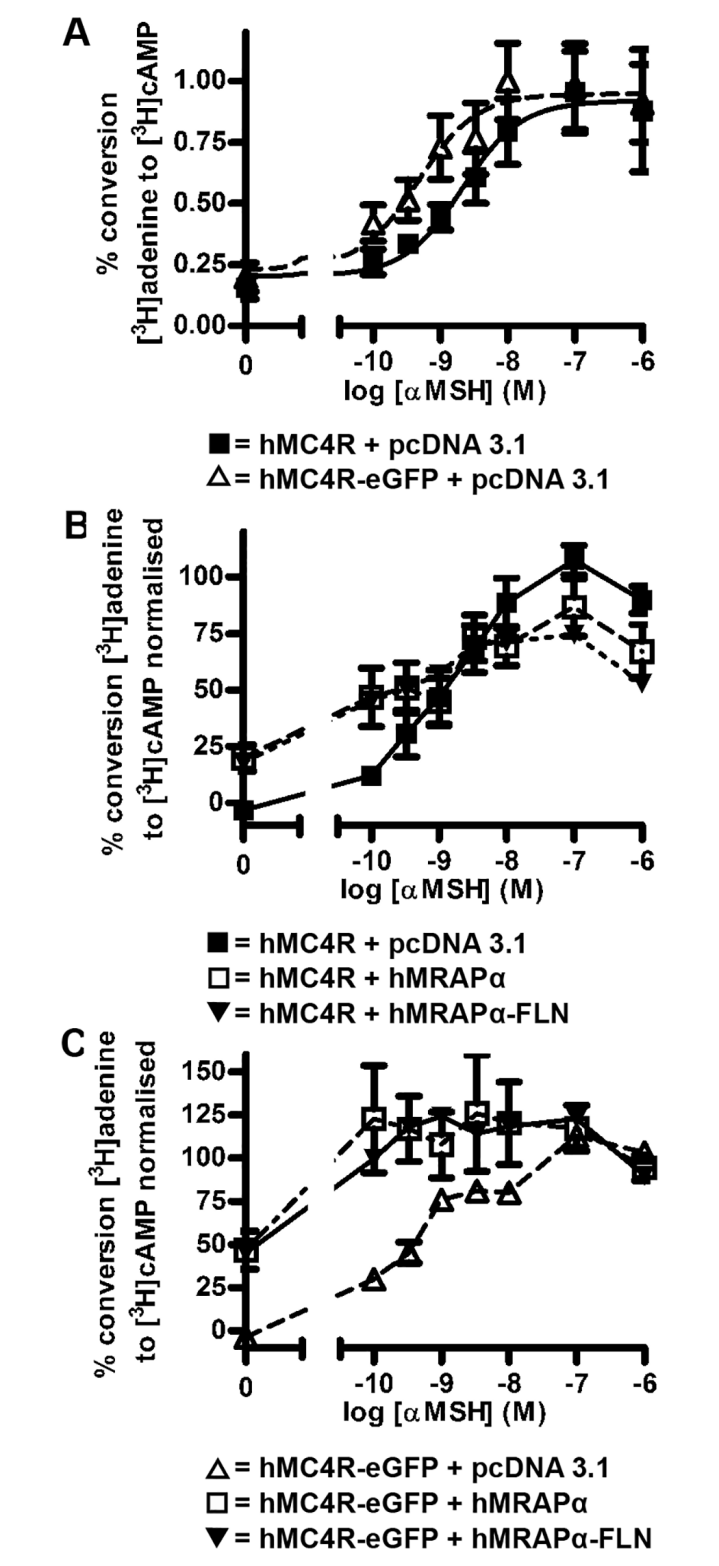
Comparison of hMC4R and hMC4R-eGFP coupling to adenylyl cyclase. hMC4R or hMC4R-eGFP was transiently co-expressed with the empty vector pcDNA 3.1 (A). hMRAPα or hMRAPα-FLN were transiently co-expressed with hMC4R (B) or hMC4R-eGFP (C). hMC4R or hMC4R-eGFP coupling to adenylyl cyclase was stimulated with increasing concentrations of α-MSH for 1 hour and adenylyl cyclase activity measured. Raw data (A) or normalised data (B and C) from at least 3 independent experiments performed in duplicate were pooled and plotted as mean ± s.e.m. Best-fit values for baseline and maximum responses and EC_50_ for these curves are shown in Table 2.

A summary of the constructs made and their purpose is provided in S1 Table. We initially constructed a tetracysteine (PG) tagged hMC4R (hMC4R-PG) for co-transfection with a variant tetracysteine (FLN) tagged hMRAPα (hMRAPα-FLN) in order for dual detection of the tetracysteine tags by biarsenical labelling with FlAsH-EDT_2_ or ReAsH-EDT_2_, respectively. The advantage of these tags is that they are small (12 amino acids) and have previously been used in conjunction with dual biarsenical labelling to study interaction of the parathyroid hormone receptor with β-arrestin 2 in live cells [12]. Although hMRAPα-FLN increased hMC4R constitutive activity similarly to hMRAPα (Fig 4 and Table 2) and had a similar subcellular localisation to hMRAPα-FLAG in HEK293 cells (S3 Fig and supplementary results), there was poor detection of ReAsH-EDT_2_ labelled hMRAPα-FLN with high background labelling in live cells (data not shown). In cells expressing hMC4R-PG labelled with FlAsH-EDT2, we observed both a poor signal to noise ratio and evidence of cytotoxicity (data not shown). Therefore both hMRAPα-FLN and hMC4R-PG were found to be unsuitable for live cell imaging.

**Table 2:** Comparison of values for hMC4R and hMC4R-eGFP coupling to adenylyl cyclase in HEK293 cells shown in Fig 4. For hMC4R or hMC4R-eGFP + pcDNA 3.1, hMRAPα or hMRAPα-FLN, normalised adenylyl cyclase data are shown as mean ± s.e.m for baseline values only. Statistically significant differences for hMC4R or hMC4R-eGFP + hMRAPα or hMRAPα-FLN were * p < 0.05. For direct comparison of hMC4R-eGFP and hMC4R coupling to adenylyl cyclase, mean values for raw data are shown ± s.e.m for baseline and maximum responses and EC_50_ values are shown with 95% confidence intervals in brackets.

Transfection	Baseline	Maximum	EC_50_ (nM)
**hMC4R + pcDNA 3.1**	-3.293 ± 2.348		
**hMC4R + hMRAPα**	19.86 ± 5.809 *		
**hMC4R + hMRAPα-FLN**	17.85 ± 6.169 *		
**hMC4R-eGFP + pcDNA 3.1**	-3.929 ± 0.9085		
**hMC4R-eGFP + hMRAPα**	46.44 ± 11.05 *		
**hMC4R-eGFP + hMRAPα-FLN**	45.20 ± 10.17 **		
**hMC4R + pcDNA 3.1**	0.204 ± 0.078	0.919 ± 0.078	2.120 (0.582, 7.715)
**hMC4R-eGFP + pcDNA 3.1**	0.233 ± 0.105	0.949 ± 0.067	0.466 (0.120, 1.811)

Fusions of GPCRs with enhanced (eGFP) are commonly used for live cell imaging [16], therefore we also generated and tested N- and C- terminal fusions of the hMC4R with eGFP. We were reluctant to tag hMRAPα with a spectral variant of eGFP because we predicted that a large fluorescent protein tag would interfere with the function of hMRAPα, which is a 19kDa transmembrane protein. However, since we found hMRAPα-FLN to function similarly to hMRAPα, we predicted that we could use this construct in combination with an eGFP tagged MC4R. hMC4R-eGFP was found to be the most suitable for live cell imaging as there were no significant differences between the baseline responses, maximum responses, or EC_50_ values for αMSH-stimulated hMC4R-eGFP coupling to adenylyl cyclase compared to untagged hMC4R (Fig 4A, Table 2). By contrast, eGFP-hMC4R showed decreased maximal coupling to adenylyl cyclase compared to untagged hMC4R (S4A Fig). Additionally, hMRAPα co-transfection with hMC4R-eGFP significantly increased baseline coupling of hMC4R-eGFP to adenylyl cyclase (with hMRAPα ~46% increase, p < 0.009, compared to baseline coupling of hMC4R-eGFP co-expressed with pcDNA 3.1 (Fig 4C, Table 2). hMRAPα co-expression with hMC4R-eGFP also enhanced 0.1–10 nM αMSH-stimulated hMC4R-eGFP coupling to adenylyl cyclase compared to hMC4R-eGFP co-expressed with pcDNA 3.1 (Fig 4C). This response did not fit a typical sigmoidal concentration response curve, and therefore only the baseline values for coupling to adenylyl cyclase were analysed. Despite eGFP-hMC4R (S4A Fig) and hMC4R-PG (S4B Fig) demonstrating altered coupling to adenylyl cyclase compared to untagged hMC4R, co-expression with either hMRAPα or hMRAPα-FLN still appeared to increase baseline coupling of both constructs to adenylyl cyclase, compared to baseline coupling of eGFP-hMC4R or hMC4R-PG expressed with pcDNA 3.1 (Supplementary results and S4C and S4D Fig). hMC4R-eGFP co-transfection with hMRAPα-FLN also significantly increased hMC4R-eGFP coupling to adenylyl cyclase (~45% increase, p < 0.009) compared to hMC4R-eGFP expression with pcDNA 3.1 (Fig 4C, Table 2).

### hMC4R-eGFP Is Observed in Highly Dynamic Vesicles Both When It Is Expressed Alone and When Expressed with hMRAPα

We initially examined hMC4R-eGFP trafficking in live HEK293 cells transiently expressing either hMC4R-eGFP alone or hMC4R-eGFP co-expressed together with hMRAPα. hMC4R-eGFP was expressed in clusters of vesicles in the cytoplasm, perinuclear region and at the plasma membrane, both when hMC4R-eGFP was transfected alone (S5A and S5C Fig) and when it was co-transfected with hMRAPα (S5B and S5D Fig). Observations of hMC4R-eGFP vesicles over time showed that they were highly dynamic, with changes in shape, position and/or brightness observed between each frame acquired at 2.2 second intervals (S5C and S5D Fig).

### Highly Dynamic hMC4R-eGFP Vesicles Are Predominantly Associated with the ER Both When hMC4R-eGFP Is Expressed Alone and When It Is Expressed with hMRAPα-FLAG

**Fig 5:**
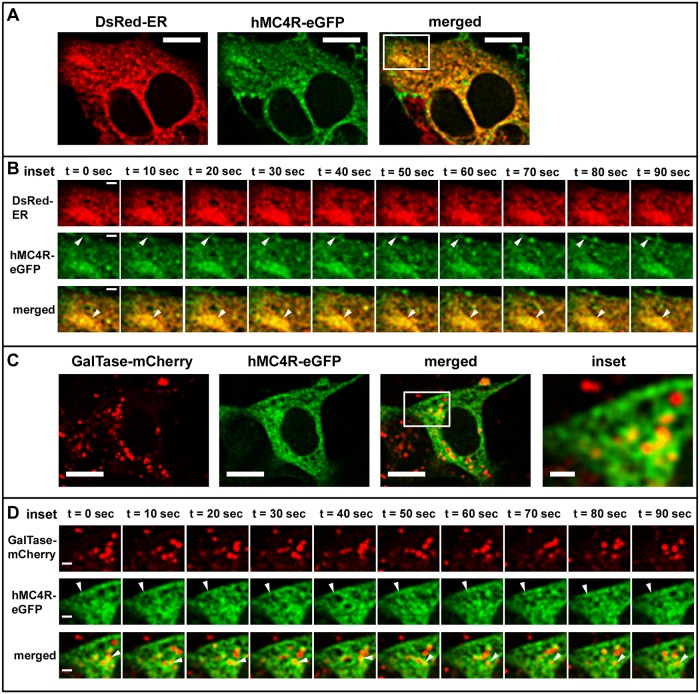
hMC4R-eGFP was predominantly observed in stable clusters of vesicles associated with a marker of the ER when expressed alone in live HEK293 cells. hMC4R-eGFP was transiently transfected into HEK293 cells stably expressing DsRed-ER (A, B) or GalTase-mCherry (C, D) and live confocal microscopy was performed with XY scans acquired every 10 seconds. hMC4R-eGFP dynamics at the cell membrane of dsRed-ER stable cells are indicated by the white arrows in the middle set of panels of sections B and D. Stable clusters containing hMC4R-eGFP and DsRed-ER or dynamic vesicles containing hMC4R-eGFP and GalTase-mCherry are indicated by the white arrows in the bottom panels of sections B and D, respectively. Scale bars = 10μm and 2μm for insets (B, D).

**Fig 6:**
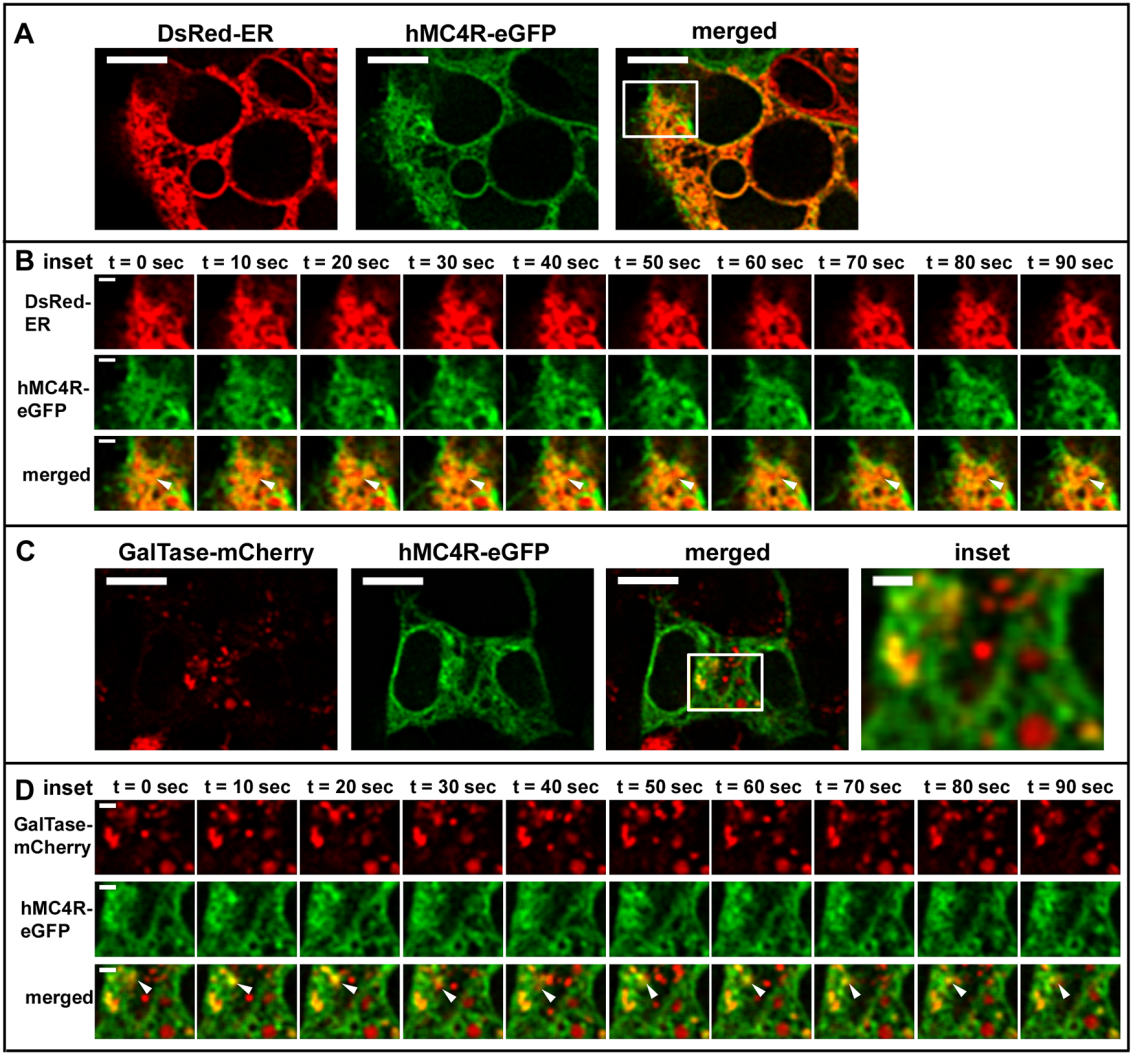
hMC4R-eGFP was predominantly observed in stable clusters of vesicles associated with marker of the ER when expressed with hMRAPα-FLAG in live HEK293 cells. hMC4R-eGFP and hMRAPα-FLAG were transiently transfected into HEK293 cells stably expressing DsRed-ER (A, B) or GalTase-mCherry (D, E) and live confocal microscopy was performed with XY scans acquired every 10 seconds. Stable clusters containing hMC4R-eGFP and DsRed-ER or dynamic vesicles containing hMC4R-eGFP and GalTase-mCherry are indicated by the white arrows in the bottom panels of sections B and D, respectively. Scale bars = 10μm and 2μm for insets (B, D).

To characterise the origin of the highly dynamic intracellular vesicles expressing hMC4R-eGFP we used confocal microscopy to examine hMC4R-eGFP trafficking in live DsRed-ER or GalTase-mCherry stable cells (Figs 5 and 6). When hMC4R-eGFP was transiently expressed alone in either DsRed-ER or GalTase-mCherry stable cells, hMC4R-eGFP fluorescent signal overlapped with DsRed-ER and GalTase-mCherry fluorescent signal (Fig 5A). hMC4R-eGFP and DsRed-ER were observed to overlap in large intracellular clusters that were relatively stable over time (Figs 5B and 6B). Highly dynamic hMC4R-eGFP vesicles which did not overlap with DsRed-ER were observed in close proximity to the plasma membrane, and appeared to change shape and/or position approximately every 10 seconds (Fig 5B and 5D, middle panels). In GalTase-mCherry stable cells, hMC4R-eGFP puncta overlapped with GalTase-mCherry puncta (Fig 5C). We observed hMC4R-eGFP and GalTase-mCherry co-localisation in small dynamic clusters of vesicles that often dissociated and re-associated between frames acquired every 10 seconds (Figs 5D and 6E, bottom panels).

To determine whether hMRAPα co-expression with hMC4R-eGFP, alters hMC4R-eGFP subcellular trafficking we performed live cell imaging of hMC4R-eGFP transiently co-transfected with hMRAPα-FLAG in HEK293 cells (Fig 6). Following live cell imaging we performed immunostaining and confocal microscopy to verify that cells expressing hMC4R-eGFP also expressed hMRAPα-FLAG (data not shown). When hMC4R-eGFP was co-transfected with hMRAPα-FLAG in DsRed-ER or GalTase-mCherry stable cells, hMC4R-eGFP signal often overlapped with signal for DsRed-ER (Fig 6A and 6B and S2B Fig) and sometimes overlapped with GalTase-mCherry signal (Fig 6C and 6D and S2B Fig).

To quantitate hMC4R-eGFP signal overlap with either dsRed-ER or GalTase-mCherry, we examined the subcellular localisation of hMC4R-eGFP stably expressed in HEK293 cells that were transiently transfected with either DsRed-ER or GalTase-mCherry. When DsRed-ER was expressed alone in hMC4R-eGFP stable cells, 31.7 ± 3% of the hMC4R-eGFP signal overlapped with DsRed-ER signal (S2B Fig). When GalTase-mCherry was transiently transfected in hMC4R-eGFP stable cells, 19.1 ± 4.5% of the hMC4R-eGFP signal overlapped with signal for GalTase-mCherry and this was significantly less than for hMC4R-eGFP signal overlap with DsRed-ER (p<0.05) (S2B Fig). When hMRAPα-FLAG was co-expressed with DsRed-ER in hMC4R-eGFP stable cells, 41.1 ± 5% of hMC4R-eGFP signal overlapped with signal for DsRed-ER and this was not significantly different from the signal overlap when hMC4R-eGFP was expressed without hMRAPα-FLAG (31.7 ± 3%) (Fig 3B). When hMRAPα-FLAG was co-expressed with GalTase-mCherry in hMC4R-eGFP stable cells, 32.2 ± 5.1% signal overlapped with signal for GalTase-mCherry and this was not significantly different from the signal overlap when hMC4R-eGFP was expressed without hMRAPα-FLAG (19.1 ± 4.5%) (S2B Fig). Overall, a significantly greater fraction of hMC4R-eGFP co-localised with DsRed-ER than with GalTase-mCherry when hMC4R-eGFP was expressed alone and when hMC4R-eGFP was expressed with hMRAPα-FLAG. hMRAPα-FLAG co-expression with hMC4R-eGFP did not result in a significant change in hMC4R-eGFP signal in the Golgi apparatus compared to the ER.

### hMC4R-eGFP Lacks Complex N-Linked Glycosylation

**Fig 7:**
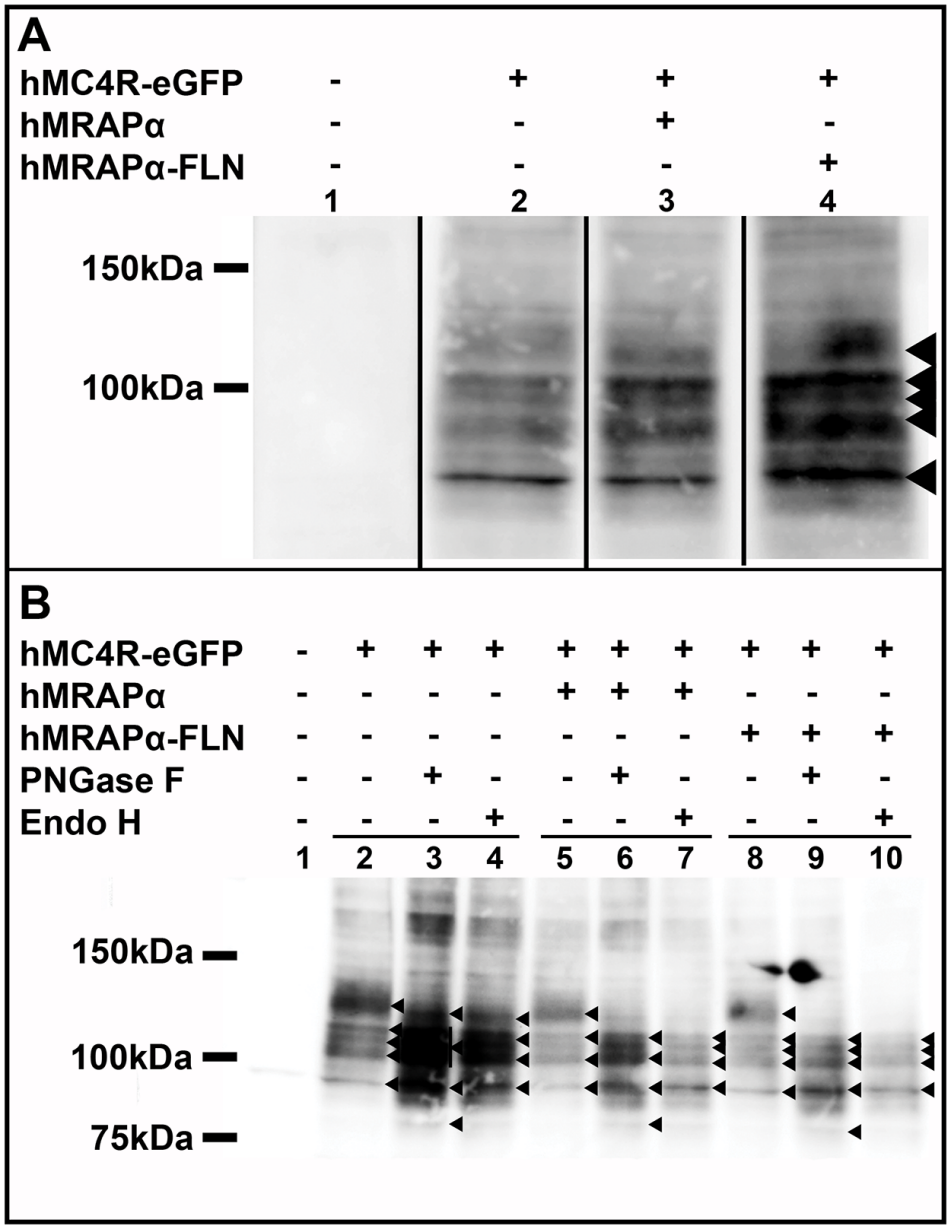
hMC4R-eGFP lacked complex N-linked glycosylation when expressed in HEK293 cells. HEK293 cells were transiently co-transfected with hMC4R-eGFP and hMRAPα, hMRAPα-FLN or the empty vector pcDNA 3.1 and western blotting was performed with anti-GFP antibody (A). Lysates for the same transfections were also treated with PNGase F and endoglycosidase H to characterise hMC4R-eGFP N-linked glycosylation (B). Molecular masses (kDa) are indicated on the left side of the western blots. Bands of interest are indicated with (◀). 1–4; 1–10, lane numbers.

hMC4R-eGFP like all of the hMC4R constructs that we have tested so far, exhibits enhanced constitutive coupling to adenylyl cyclase when co-expressed with hMRAPα (Fig 4C). We previously showed that hMRAPα not only enhanced hMC4R constitutive coupling to adenylyl cyclase but it also altered HA-hMC4R molecular mass and complex N-linked glycosylation which is added in the Golgi apparatus [9]. Since bigger fractions of both HA-hMC4R and hMC4R-eGFP colocalised with DsRed-ER (48.0 ± 0.04 and 31.7 ± 0.03%, respectively) compared with GalTase-mCherry (28.7 ± 0.04 and 19.1 ± 0.03%, respectively), we determined whether hMRAPα altered hMC4R-eGFP maturation similar to HA-hMC4R. We therefore characterised hMC4R-eGFP molecular mass and complex glycosylation following co-expression with hMRAPα or hMRAPα-FLN (Fig 4C). hMC4R-eGFP was observed as five bands of ~90 kDa, ~100 kDa, ~110kDa, ~115 kDa and ~130 kDa following co-expression with pcDNA 3.1 (Fig 7A, lane 2). These bands are all larger than the predicted molecular mass of hMC4R-eGFP (63kDa; hMC4R is ~36kDa and eGFP is ~27kDa). These same five bands were also observed following co-expression of hMC4R-eGFP with either hMRAPα (Fig 7A, lane 3) or hMRAPα-FLN (Fig 7A, lane 4), indicating that hMRAPα co-expression did not induce a change in hMC4R-eGFP molecular mass.

We examined hMC4R-eGFP N-linked glycosylation with specific endoglycosidases. In the absence of treatment with endoglycosidases, hMC4R-eGFP co-expressed with pcDNA 3.1, hMRAPα or hMRAPα-FLN was observed as five bands of ~90 kDa, ~100 kDa, ~110 kDa, ~115 kDa and ~130 kDa (Fig 7B, lanes 2, 5, and 8). When hMC4R-eGFP co-expressed with pcDNA 3.1 was digested with PNGase F, the ~130 kDa hMC4R-eGFP specific band was shifted slightly downwards to ~120 kDa, a large smear was observed from ~100–115 kDa and an additional band was observed at ~80 kDa, which was not observed for the undigested hMC4R-eGFP (Fig 7B, lane 2 vs. lane 3). The smear from ~100–115 kDa appears to consist of three bands at ~100 kDa, ~110 kDa and ~115 kDa, as was also observed for hMC4R-eGFP co-expressed with hMRAPα or hMRAPα-FLN and digested with PNGase F (Fig 7B, lanes 6 and 9). When hMC4R-eGFP co-expressed with pcDNA 3.1 was digested with endoglycosidase H, the ~130 kDa hMC4R-eGFP specific band was again shifted slightly downwards to ~120 kDa, but this time two bands were observed at ~100 kDa and ~115 kDa and the ~80 kDa band present in the PNGase F digest was not observed (Fig 7B, lane 4). These same four bands were observed for hMC4R-eGFP co-expressed with hMRAPα and hMRAPα-FLN and digested with endoglycosidase H (Fig 7B, lanes 7 and 10). Overall a similar pattern of bands was observed for hMC4R-eGFP co-expressed with hMRAPα or hMRAPα-FLN and digested with PNGase F or endoglycosidase H (Fig 7B, lanes 6, 7, 9, 10). The ~130kDa hMC4R-eGFP specific band was sensitive to both PNGase F and endoglycosidase H digestion, and therefore represented hMC4R-eGFP with high mannose oligosaccharides. There were no hMC4R-eGFP specific bands that were sensitive to PNGase F and resistant to endoglycosidase H, indicating that the hMC4R-eGFP lacks complex N-linked glycosylation, both with and without hMRAPα co-expression.

## Discussion

Here we provide new evidence that hMRAPα and hMRAP2 may have divergent effects on the function of the hMC4R. hMRAPα-FLAG, but not hMRAP2-FLAG increased hMC4R constitutive activity when expressed together with the hMC4R in HEK293 cells. This effect was observed for all the different tagged combinations of hMC4R and hMRAPα that we tested, therefore hMRAPα enhancement of hMC4R constitutive activity is robust. We also did not observe hMRAP2-FLAG to decrease hMC4R constitutive activity, however we did observe both hMRAPα-FLAG and hMRAP2-FLAG to significantly increase HA-hMC4R sensitivity to α-MSH and HA-hMC4R maximal coupling to adenylyl cyclase. Our hMRAP2-FLAG construct was correctly expressed in HEK293 cells, as we observed a similar hMRAP2-FLAG expression pattern to what has been previously published for hMRAP2-FLAG in Chinese Hamster Ovary (CHO) cells [2].

Our data for MRAPα and MRAP2, and those of others, point to potential species differences for MRAP function. Our data for hMC4R constitutive activity contrast with previous studies showing that MRAP2 mouse and zebrafish homologues decrease MC4R constitutive activity [4, 5]. However, our observation that hMRAP2 sensitises HA-hMC4R coupling to Gαs reflects previous studies on mMRAP2 and zMRAP2b [5]. In our hands this effect is peculiar to the FLAG and HA epitope combination on hMRAP2 and hMC4R respectively. The differences between our data and those of Sebag *et al*. may reflect their use of a Cre luciferase assay to measure MC4R signalling, their use of Chinese Hamster Ovary (CHO) cells as an expression system and/or their transfection of MRAP2 at a higher ratio to MC4R compared to the 1:1 ratio used in our study. Alternatively the data could collectively indicate that while there is extensive sequence conservation at least between mouse and human MRAP proteins, there could still be differences in mouse, zebrafish and human MRAP functional effects on the MC4R. Given the potential implications for hMRAP proteins in modifying hMC4R function in the regulation of appetite and body weight, we believe that it is vital that further *in vitro* studies on MRAPα or MRAP2 function be performed using a human system.

We have extended our previous work showing that hMRAPα-FLAG alters HA-hMC4R molecular mass and complex N-linked glycosylation by demonstrating that co-expression with hMRAPα does not appear to have any major effects on hMC4R secretory trafficking [9]. We show here that hMRAPα-FLAG co-expression does not alter either HA-hMC4R or hMC4R-eGFP partitioning into the ER or Golgi apparatus. This suggests that hMRAPα does not alter hMC4R complex N-linked glycosylation by altering hMC4R trafficking between the ER and the plasma membrane. An alternative hypothesis is that hMRAPα interacts with the hMC4R in one or both of these compartments, resulting in altered hMC4R complex N-linked glycosylation.

We also demonstrate for the first time that an hMRAPα-induced change in hMC4R complex N-linked glycosylation may not be required for the hMRAPα to increase hMC4R constitutive activity at the plasma membrane. Both HA-hMC4R and hMC4R-eGFP exhibit increased constitutive activity following co-expression with hMRAPα. In our previous study we showed that HA-hMC4R has complex N-linked glycosylation, which is altered by hMRAPα-FLAG co-expression [9]. In the current study we show that hMC4R-eGFP lacks complex N-linked glycosylation. hMC4R-eGFP complex N-linked glycosylation cannot be altered by co-expression with hMRAPα, therefore hMRAPα enhancement of hMC4R constitutive activity is not dependent on hMC4R complex glycosylation.

Interestingly, hMRAPα enhanced hMC4R constitutive activity was greater for hMC4R-eGFP compared to untagged hMC4R and the other tagged hMC4R constructs that we examined. This may reflect a difference in the conformational state of hMC4R-eGFP compared to the other constructs. Changes in conformational state have been associated with modulation of GPCR constitutive activity [17, 18]. For example, hMC4R-eGFP may exhibit a more stable interaction with Gαs than HA-hMC4R in the basal state. Since hMRAPα cannot influence hMC4R-eGFP complex N-linked glycosylation, we hypothesise that hMRAPα may promote a conformational change in hMC4R-eGFP through interactions in either the ER or Golgi apparatus, resulting in increased hMC4R constitutive activity. This may involve residues in the N-terminus, the second, third, fourth and sixth TM domains and the third ICL of the hMC4R, as all of these regions have been shown to be involved in MC4R constitutive activity [6, 19–21].

Importantly, our study shows that different hMC4R recombinant proteins can produce similar signalling responses, while possessing different post-translational modifications. hMC4R-eGFP has been widely used to study MC4R function and to screen MC4R pharmacological agonists and antagonists [22] and yet this construct may not behave like endogenous hMC4R. We predict that HA-hMC4R mimics the native receptor more closely than hMC4R-eGFP since the 3x HA tag is much smaller than the eGFP tag. While hMC4R-eGFP is not an ideal model for studying hMC4R function, there currently is no alternative to using hMC4R-eGFP for live cell imaging. Our study highlights the need for better technologies for the detection of intracellular proteins in live cell imaging studies to enable more detailed and accurate analysis of protein-protein interactions.

The physiological significance of hMRAPα enhancement of hMC4R constitutive activity will only be realised if hMC4R and hMRAPα interact *in vivo*. There is potential for this interaction since hMRAPα and hMC4R mRNA are both detected in human brain, adrenal gland, adipose tissue, testes, ovary and heart [6]. One might expect that the examination of phenotypic differences between patients with Familial Glucocorticoid Deficiency (FGD) type I and type II could identify additional physiological effects of hMRAPα. However the phenotype of FGD patients is complicated because they require treatment with glucocorticoids from birth to survive. Additionally FGD is a very rare disorder, and therefore novel phenotypes may be difficult to identify. One obese patient with an *MRAP1* mutation has been identified, but it was not determined whether his obesity was the result of a defect in neural melanocortin receptor signalling [23]. Interestingly, a physiological role for MRAP2 in energy homeostasis was identified when loss of mouse MRAP2 function resulted in obesity [4]. Similarly to *MRAP1*, evidence for *MRAP2* mutations associated with human obesity is limited. Four rare heterozygous variants in 1000 genomes from severely obese individuals were found and only one variant was clearly disruptive [4]. This indicates that if *MRAP2* mutations contribute to human obesity, they do so rarely [4].

Regardless of the physiological significance, understanding the mechanism(s) underlying hMRAPα enhanced hMC4R constitutive activity could be of major significance in the development of therapies for diseases caused by defective hMC4R. First, there are ‘hot spots’ in the hMC4R N-terminus, third intracellular loop and in some of the hMC4R transmembrane domains where mutations associated with obesity either increase or decrease constitutive activity. It is currently not understood how mutations that increase hMC4R constitutive activity can cause obesity. We propose that hMRAPα co-expression with the hMC4R is a useful tool to explore mechanism(s) of hMC4R constitutive activity and their physiological significance. Second, the majority of hMC4R point mutations associated with obesity result in defective hMC4R that are retained intracellularly and have impaired signalling responses [22, 24, 25]. Augmentation of hMC4R signalling in obese patients with defective hMC4R could be achieved through enhancement of hMC4R cell surface expression and/or the identification of positive allosteric modulators of hMC4R functional activity [22, 26, 27]. Future studies investigating how hMRAPα enhances hMC4R constitutive activity could therefore lead to the development of new therapeutic interventions for obesity and metabolic diseases.

## Supporting Information

S1 Table Summary of constructs, their purpose and outcomes of functional testing. (PDF)

S1 Text Supplementary Methods and Results. Imaging of biarsenically labeled HEK293 cells. Evaluation of hMRAPα-FLN for live cell imaging.
(PDF)

S1 Fig Comparison of hMRAPα and hMRAP2 effects on hMC4R coupling to adenylyl cyclase. hMC4R was transiently co-transfected with pcDNA 3.1, hMRAPα or hMRAP2. hMC4R coupling to adenylyl cyclase was stimulated with increasing concentrations of α-MSH for 1 hour and adenylyl cyclase activity measured. Normalised data from 1 experiment performed in duplicate was plotted as mean ± s.e.m. Values for baseline coupling of hMC4R coupling to adenylyl cyclase with and without co-expression with hMRAP2 are shown in Table 1.
(TIF)

S2 Fig Greater fractions of HA-hMC4R and hMC4R-eGFP observed in ER than in the Golgi apparatus. (A) A significantly greater fraction of HA-hMC4R fluorescence overlapped with fluorescent signal for DsRed-ER compared to fluorescent signal for GalTase-mCherry, both when HA-hMC4R was expressed alone and when HA-hMC4R was co-expressed with hMRAPα-FLAG. (B) A significantly greater fraction of hMC4R-eGFP fluorescence overlapped with fluorescent signal for DsRed-ER compared to fluorescent signal for GalTase-mCherry when hMC4R-eGFP was expressed alone, but not when hMC4R-eGFP was co-expressed with hMRAPα-FLAG. M co-efficients for the overlap of HA-hMC4R or hMC4R-eGFP fluorescent signal are presented as the mean ± SEM. Significant differences were determined using one-way ANOVA and Tukey’s post-hoc test. *, p<0.05; **, p<0.01; ***, p<0.001.
(TIF)

S3 Fig hMRAPα-FLN and hMRAPα-FLAG had similar subcellular localisation when expressed in HEK293 cells. hMRAPα-FLN (A, D, G), hMRAPα-FLAG-FLN (B, E, H) or hMRAPα-FLAG (C, F, I) were transiently transfected into HEK293 cells, labeled with 2.5μM ReAsH-EDT_2_ and anti-FLAG antibody, and confocal microscopy was performed. Scale bars = 10μM.
(TIF)

S4 Fig Comparison of hMC4R, eGFP-hMC4R and hMC4R-PG coupling to adenylyl cyclase. eGFP-hMC4R (A) or hMC4R-PG (B) were transiently transfected in parallel with hMC4R or transiently co-transfected with pcDNA 3.1, hMRAPα or hMRAPα-FLN (C, D). hMC4R, eGFP-hMC4R or hMC4R-PG coupling to adenylyl cyclase was stimulated with increasing concentrations of α-MSH for 1 hour and adenylyl cyclase activity measured. hMC4R, eGFP-hMC4R (A) or hMC4R-PG (B) was transiently co-expressed with the empty vector pcDNA 3.1. hMRAPα or hMRAPα-FLN was transiently co-expressed with eGFP-hMC4R (C) or hMC4R-PG (D). hMC4R, hMC4R-PG and eGFP-hMC4R coupling to adenylyl cyclase were stimulated with increasing concentrations of α-MSH for 1 hour and adenylyl cyclase activity measured. Raw data from 1 experiment performed in duplicate was plotted as mean ± s.e.m. Normalised data from 1 experiment (D) performed in duplicate was plotted as mean ± s.e.m.
(TIF)

S5 Fig hMC4R-eGFP trafficking was highly dynamic in live HEK293 cells, both when hMC4R-eGFP was expressed alone, or when expressed with hMRAPα. HEK293 cells were transiently transfected with hMC4R-eGFP with pcDNA 3.1 (A, C) or hMRAPα (B, D) and live confocal microscopy was performed with XY scans acquired approximately every 2.2 seconds. Movement of vesicles containing hMC4R-eGFP expression (indicated by white arrows in insets C and D) was observed over time. Scale bars = 10μm, 2μm for insets.
(TIF)
