# Supplementary material for: hMRAPα, but Not hMRAP2, Enhances hMC4R Constitutive Activity in HEK293 Cells and This Is Not Dependent on hMRAPα Induced Changes in hMC4R Complex N-linked Glycosylation
Source: PLoS One. 2015 Oct 15;10(10):e0140320. doi: 10.1371/journal.pone.0140320 (PMC4607451; doi:10.1371/journal.pone.0140320)
Supplement: S1 Table — (PDF) [file pone.0140320.s001.pdf]

Construct	Purpose for developing construct	Outcome
hMRAPα	Functional comparison of untagged and tagged hMRAPα	Good for functional testing
hMRAPα-FLAG	Fixed confocal microscopy analysis of hMRAPα subcellular localisation	Good for functional testing and fixed confocal microscopy
hMRAPα-FLAG-FLN	Fixed confocal microscopy to determine that hMRAPα-FLN subcellular localisation is similar to hMRAPα-FLAG	Good for fixed confocal microscopy
hMRAPα-FLN	Dual biarsenical imaging of hMRAPα and hMC4R	Good for functional testing but not for biarsenical imaging
hMC4R	Functional comparison of untagged and tagged hMC4R	Good for functional testing
HA-hMC4R	Fixed confocal microscopy analysis of hMC4R subcellular localisation	Good for functional testing and fixed confocal microscopy
hMC4R-PG	Dual biarsenical imaging of hMC4R and hMRAPα	Not good for functional testing nor for biarsenical imaging
hMC4R-eGFP	Live cell imaging of hMC4R	Good for functional testing and live cell imaging although subcellular localisation differs from HA-hMC4R
eGFP-hMC4R	Live cell imaging of hMC4R	Not good for functional testing

S1 Table: Summary of constructs, their purpose and outcomes of functional testing.
