# Supplementary material for: hMRAPα, but Not hMRAP2, Enhances hMC4R Constitutive Activity in HEK293 Cells and This Is Not Dependent on hMRAPα Induced Changes in hMC4R Complex N-linked Glycosylation
Source: PLoS One. 2015 Oct 15;10(10):e0140320. doi: 10.1371/journal.pone.0140320 (PMC4607451; doi:10.1371/journal.pone.0140320)
Supplement: S1 Text — Imaging of biarsenically labeled HEK293 cells. Evaluation of hMRAPα-FLN for live cell imaging. (PDF) [file pone.0140320.s002.pdf]

Supplementary Information

Methods

Imaging of biarsenically labeled HEK293 cells

HEK293 cells were seeded into 35 mm fluorodishes or coverslips in 24 well plates prepared as for immunocytochemistry. Forty-eight hours later when the cells were 30-50% confluent, they were transiently transfected with hMRAPα-FLN, hMRAPα-FLAG-FLN or hMRAPa-FLAG. After a further 48 hours, the media was removed and the cells were washed 3x with warm Hanks Balanced Salt Solution (HBSS) (Invitrogen, Carlsbad, CA, USA) + 500g/L glucose + 25mM HEPES. Transfected cells were then labeled with 2.5μM ReAsH-EDT_2 (diluted in HBSS + 500 g/L glucose + 25mM HEPES) for 1 hour at 37°C, under 5% CO_2. The cells were then washed briefly 2 x with warm HBSS + 1g/L glucose + 25mM HEPES + 250μM 2,3-dimercapto-1-propanol (BAL) (Merck KGaA, Darmstadt, Germany) followed by a 10 minute incubation at 37°C to remove unbound ReAsH-EDT_2. For detection of hMRAPα-FLAG-FLN or hMRAPα-FLAG, transfected cells were fixed and labeled with anti-FLAG antibody as described in the main text. Z-stack images at 1μm intervals were acquired with a 60x oil immersion lens (numerical aperture = 1.3), with 3x optical zoom. Maximal intensity z-projections were processed in Image J [1].

Results:

Evaluation of hMRAPα-FLN for live cell imaging

We evaluated hMRAP-FLN for potential live cell imaging together with hMC4R-PG or hMC4R-eGFP. We show that the subcellular localisation of hMRAPα-FLN was similar to that of hMRAPα-FLAG, which we had used for our previous studies. We generated hMRAPα-FLAG-FLN to enable comparison of tetracysteine and FLAG tagged hMRAPα subcellular localisation following anti-FLAG immunostaining. hMRAPα-FLN, hMRAPα-FLAG-FLN and hMRAPα-FLAG were each transiently expressed in HEK293 cells and the cells were labelled with ReAsH-EDT_2 and anti-FLAG antibody. hMRAPα-FLN was specifically detected with ReAsH-EDT_2, but not with anti-FLAG

antibody (Fig. S3A, D and G). hMRAPα-FLAG was detected with anti-FLAG antibody but not with ReAsH-EDT$_2$ (Fig. S3C, F and I). hMRAPα-FLAG-FLN was detected with both ReAsH-EDT$_2$ and anti-FLAG antibody (Fig. S3B, E and H). hMRAPα-FLN (Fig. S3G), hMRAPα-FLAG-FLN (Fig. S3H) and hMRAPα-FLAG (Fig. S3I) were all observed in the perinuclear region, cytoplasm and plasma membrane of HEK293 cells.

We also evaluated the function of both hMC4R-PG and hMRAPα-FLN for effects on adenylyl cyclase activation compared to the untagged hMC4R and hMRAPα respectively. hMC4R-PG exhibited increased α-MSH stimulated maximal coupling to adenylyl cyclase compared to untagged hMC4R (Fig. S4B).

Either hMRAPα or hMRAPα-FLN co-expression with hMC4R significantly increased baseline coupling of the hMC4R to adenylyl cyclase (with hMRAPα ~20% increase, $p < 0.05$; with hMRAPα-FLN ~18% increase, $p < 0.05$) compared to baseline coupling of hMC4R expressed with pcDNA 3.1 (Fig. 4C, Table 1). Either hMRAPα or hMRAPα-FLN co-expression also appeared to increase eGFP-hMC4R (Fig. S4C) or hMC4R-PG (Fig. S4D) baseline coupling to adenylyl cyclase, compared to baseline coupling of eGFP-hMC4R or hMC4R-PG expressed with pcDNA 3.1.

Reference

[1] Rasband, W. S. (1997-2011) Image J, In http://imagej.nih.gov/ij/.

Figure Legends

S1 Fig. Comparison of hMRAPα and hMRAP2 effects on hMC4R coupling to adenylyl cyclase. hMC4R was transiently co-transfected with pcDNA 3.1, hMRAPα or hMRAP2. hMC4R coupling to adenylyl cyclase was stimulated with increasing concentrations of α-MSH for 1 hour and adenylyl cyclase activity measured. Normalised data from 1 experiment performed in duplicate was plotted as

mean ± s.e.m. Values for baseline coupling of hMC4R coupling to adenylyl cyclase with and without co-expression with hMRAP2 are shown in Table 1.

S2 Fig. **(A)** A significantly greater fraction of HA-hMC4R fluorescence overlapped with fluorescent signal for DsRed-ER compared to fluorescent signal for GalTase-mCherry, both when HA-hMC4R was expressed alone and when HA-hMC4R was co-expressed with hMRAPα-FLAG. **(B)** A significantly greater fraction of hMC4R-eGFP fluorescence overlapped with fluorescent signal for DsRed-ER compared to fluorescent signal for GalTase-mCherry when hMC4R-eGFP was expressed alone, but not when hMC4R-eGFP was co-expressed with hMRAPα-FLAG. M co-efficients for the overlap of HA-hMC4R or hMC4R-eGFP fluorescent signal are presented as the mean ± SEM. Significant differences were determined using one-way ANOVA and Tukey's post-hoc test. *, $p<0.05$; **, $p<0.01$; ***, $p<0.001$.

S3 Fig. hMRAPα-FLN and hMRAPα-FLAG had similar subcellular localisation when expressed in HEK293 cells. hMRAPα-FLN (A, D, G), hMRAPα-FLAG-FLN (B, E, H) or hMRAPα-FLAG (C, F, I) were transiently transfected into HEK293 cells, labeled with 2.5μM ReAsH-EDT_2 and anti-FLAG antibody, and confocal microscopy was performed. Scale bars = 10μM.

S4 Fig. Comparison of hMC4R, eGFP-hMC4R and hMC4R-PG coupling to adenylyl cyclase. eGFP-hMC4R (A) or hMC4R-PG (B) were transiently transfected in parallel with hMC4R or transiently co-transfected with pcDNA 3.1, hMRAPα or hMRAPα-FLN (C, D). hMC4R, eGFP-hMC4R or hMC4R-PG coupling to adenylyl cyclase was stimulated with increasing concentrations of α-MSH for 1 hour and adenylyl cyclase activity measured. hMC4R, eGFP-hMC4R (A) or hMC4R-PG (B) was transiently co-expressed with the empty vector pcDNA 3.1. hMRAPα or hMRAPα-FLN was transiently co-expressed with eGFP-hMC4R (C) or hMC4R-PG (D). hMC4R, hMC4R-PG and

eGFP-hMC4R coupling to adenylyl cyclase were stimulated with increasing concentrations of α-MSH for 1 hour and adenylyl cyclase activity measured. Raw data from 1 experiment performed in duplicate was plotted as mean ± s.e.m. Normalised data from 1 experiment (D) performed in duplicate was plotted as mean ± s.e.m.

S5 Fig. hMC4R-eGFP trafficking was highly dynamic in live HEK293 cells, both when hMC4R-eGFP was expressed alone, or when expressed with hMRAPα. HEK293 cells were transiently transfected with hMC4R-eGFP with pcDNA 3.1 (A, C) or hMRAPα (B, D) and live confocal microscopy was performed with XY scans acquired approximately every 2.2 seconds. Movement of vesicles containing hMC4R-eGFP expression (indicated by white arrows in insets C and D) was observed over time. Scale bars = 10μm, 2μm for insets.
