# Supplementary figures and images for: hMRAPα, but Not hMRAP2, Enhances hMC4R Constitutive Activity in HEK293 Cells and This Is Not Dependent on hMRAPα Induced Changes in hMC4R Complex N-linked Glycosylation
Source: PLoS One. 2015 Oct 15;10(10):e0140320. doi: 10.1371/journal.pone.0140320 (PMC4607451; doi:10.1371/journal.pone.0140320)

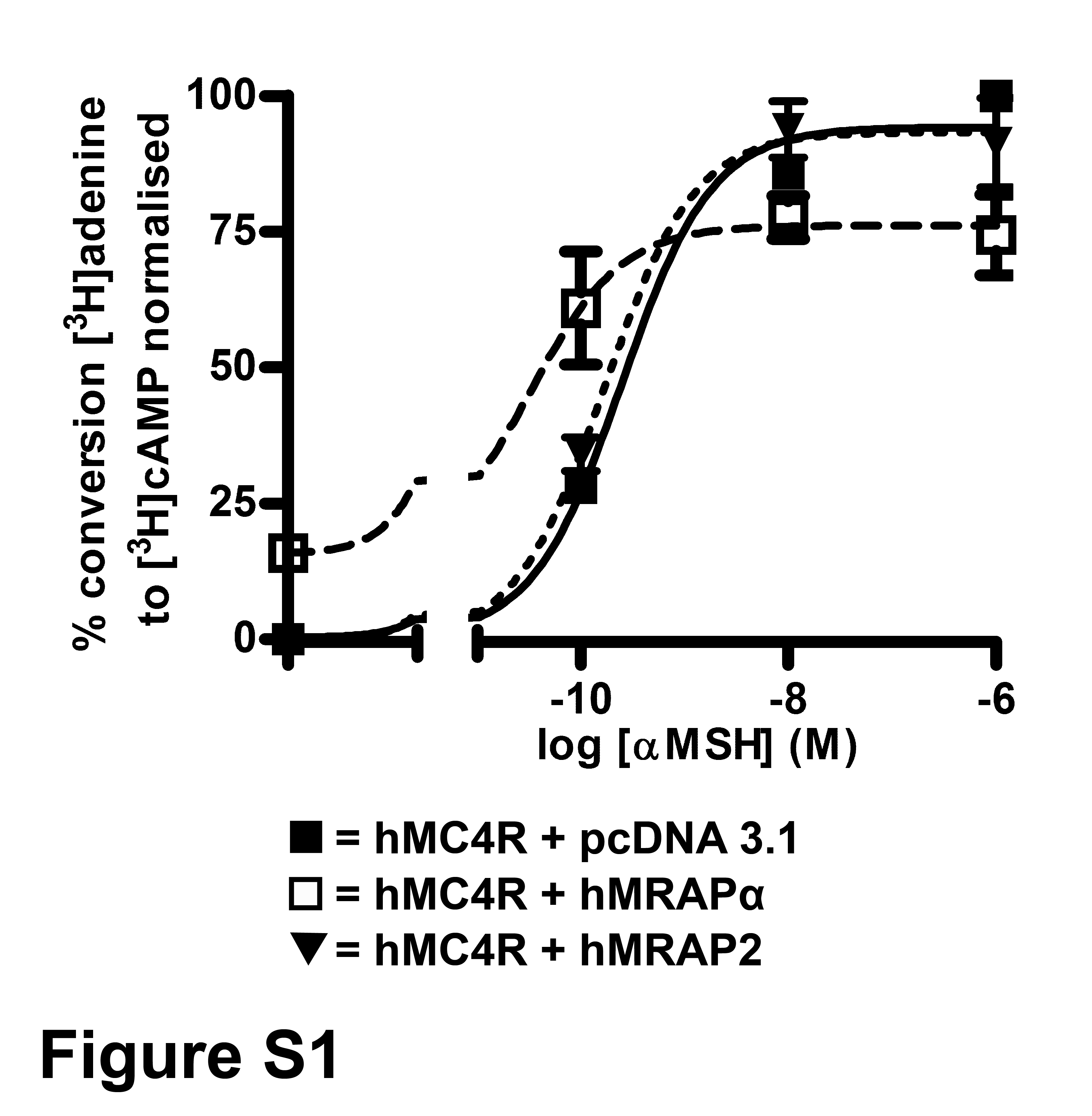

Supplement: S1 Fig — hMC4R was transiently co-transfected with pcDNA 3.1, hMRAPα or hMRAP2. hMC4R coupling to adenylyl cyclase was stimulated with increasing concentrations of α-MSH for 1 hour and adenylyl cyclase activity measured. Normalised data from 1 experiment performed in duplicate was plotted as mean ± s.e.m. Values for baseline coupling of hMC4R coupling to adenylyl cyclase with and without co-expression with hMRAP2 are shown in Table 1. (TIF) [file pone.0140320.s003.tif]

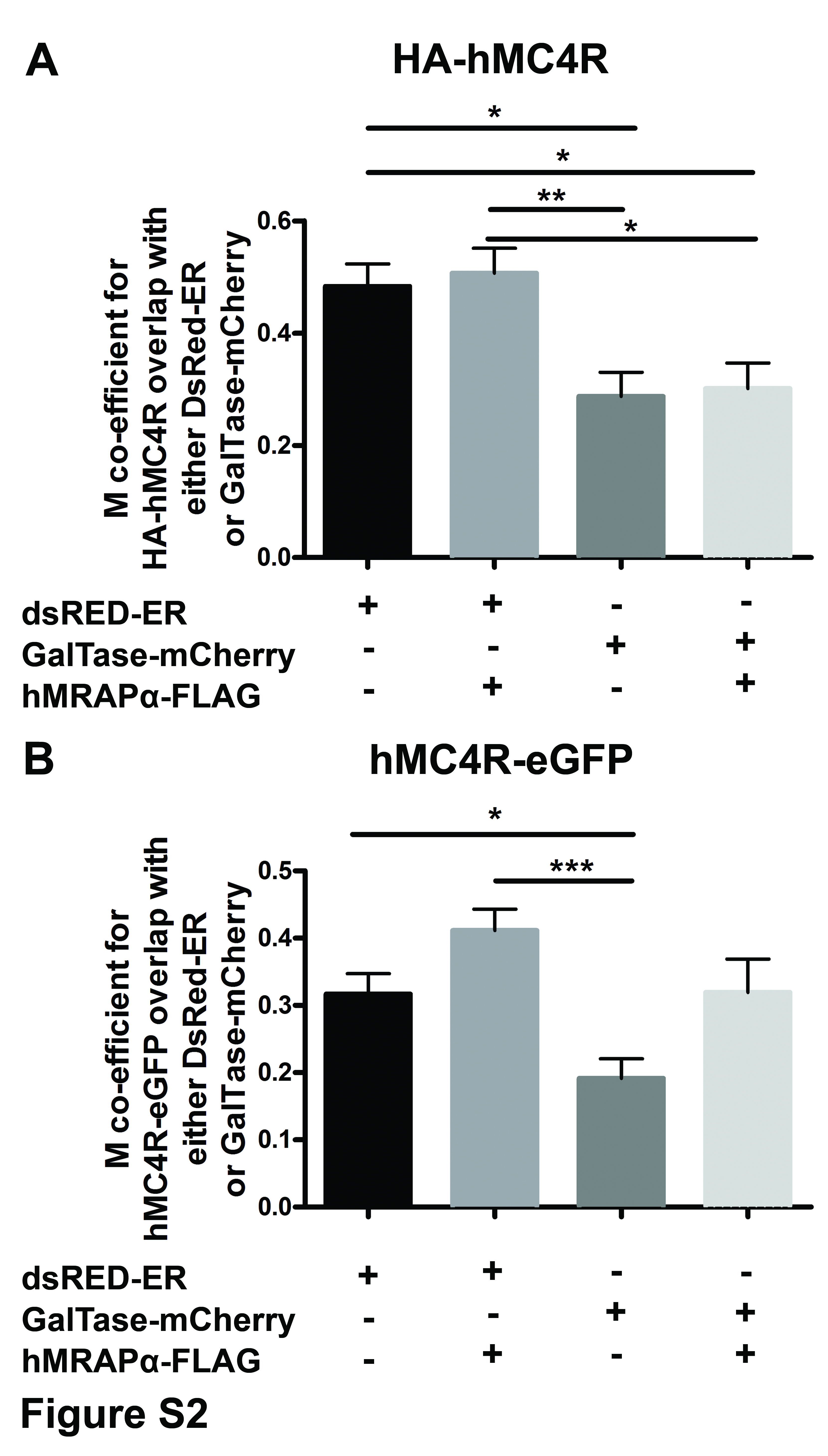

Supplement: S2 Fig — (A) A significantly greater fraction of HA-hMC4R fluorescence overlapped with fluorescent signal for DsRed-ER compared to fluorescent signal for GalTase-mCherry, both when HA-hMC4R was expressed alone and when HA-hMC4R was co-expressed with hMRAPα-FLAG. (B) A significantly greater fraction of hMC4R-eGFP fluorescence overlapped with fluorescent signal for DsRed-ER compared to fluorescent signal for GalTase-mCherry when hMC4R-eGFP was expressed alone, but not when hMC4R-eGFP was co-expressed with hMRAPα-FLAG. M co-efficients for the overlap of HA-hMC4R or hMC4R-eGFP fluorescent signal are presented as the mean ± SEM. Significant differences were determined using one-way ANOVA and Tukey’s post-hoc test. *, p<0.05; **, p<0.01; ***, p<0.001. (TIF) [file pone.0140320.s004.tif]

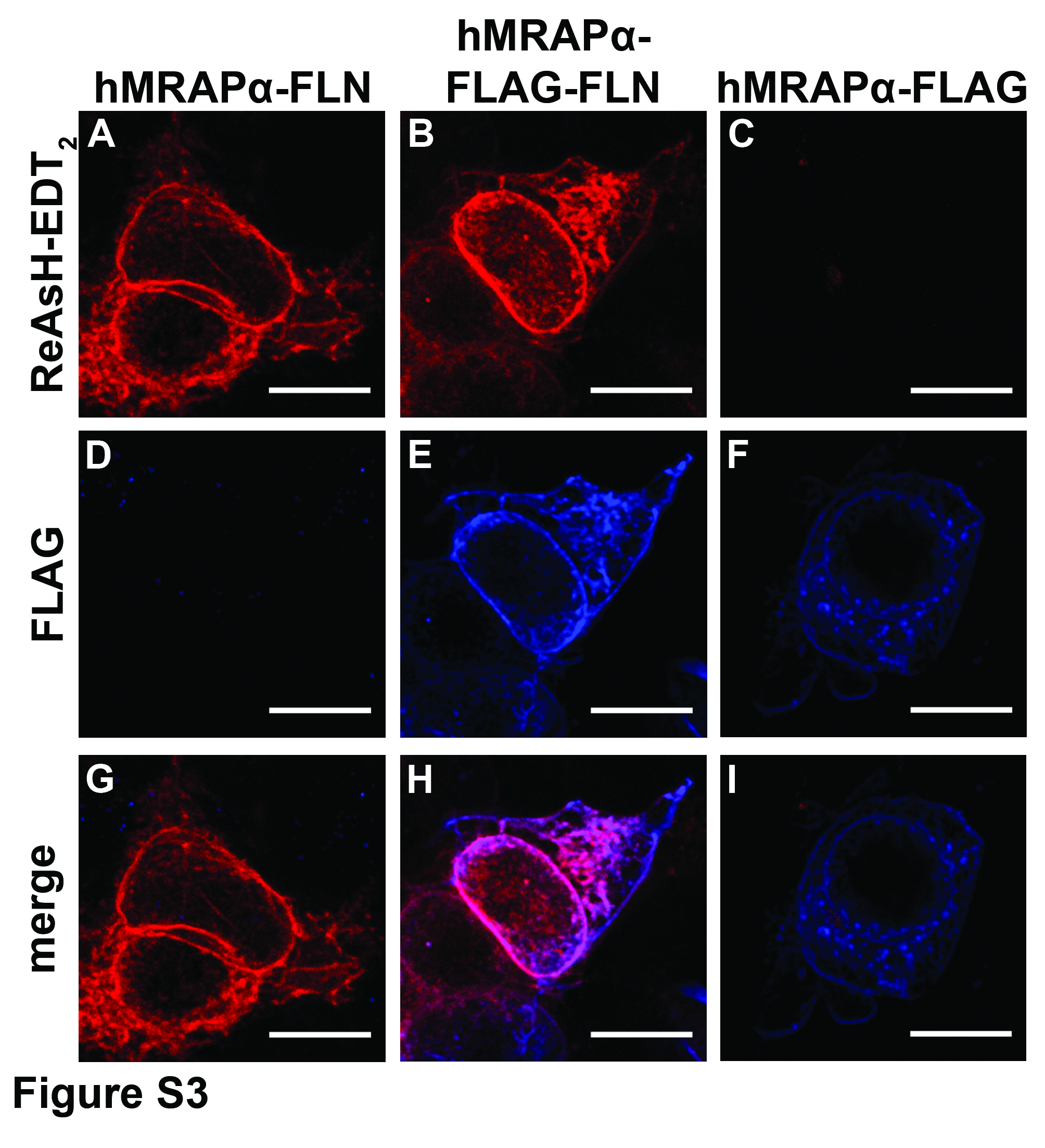

Supplement: S3 Fig — hMRAPα-FLN (A, D, G), hMRAPα-FLAG-FLN (B, E, H) or hMRAPα-FLAG (C, F, I) were transiently transfected into HEK293 cells, labeled with 2.5μM ReAsH-EDT2 and anti-FLAG antibody, and confocal microscopy was performed. Scale bars = 10μM. (TIF) [file pone.0140320.s005.tif]

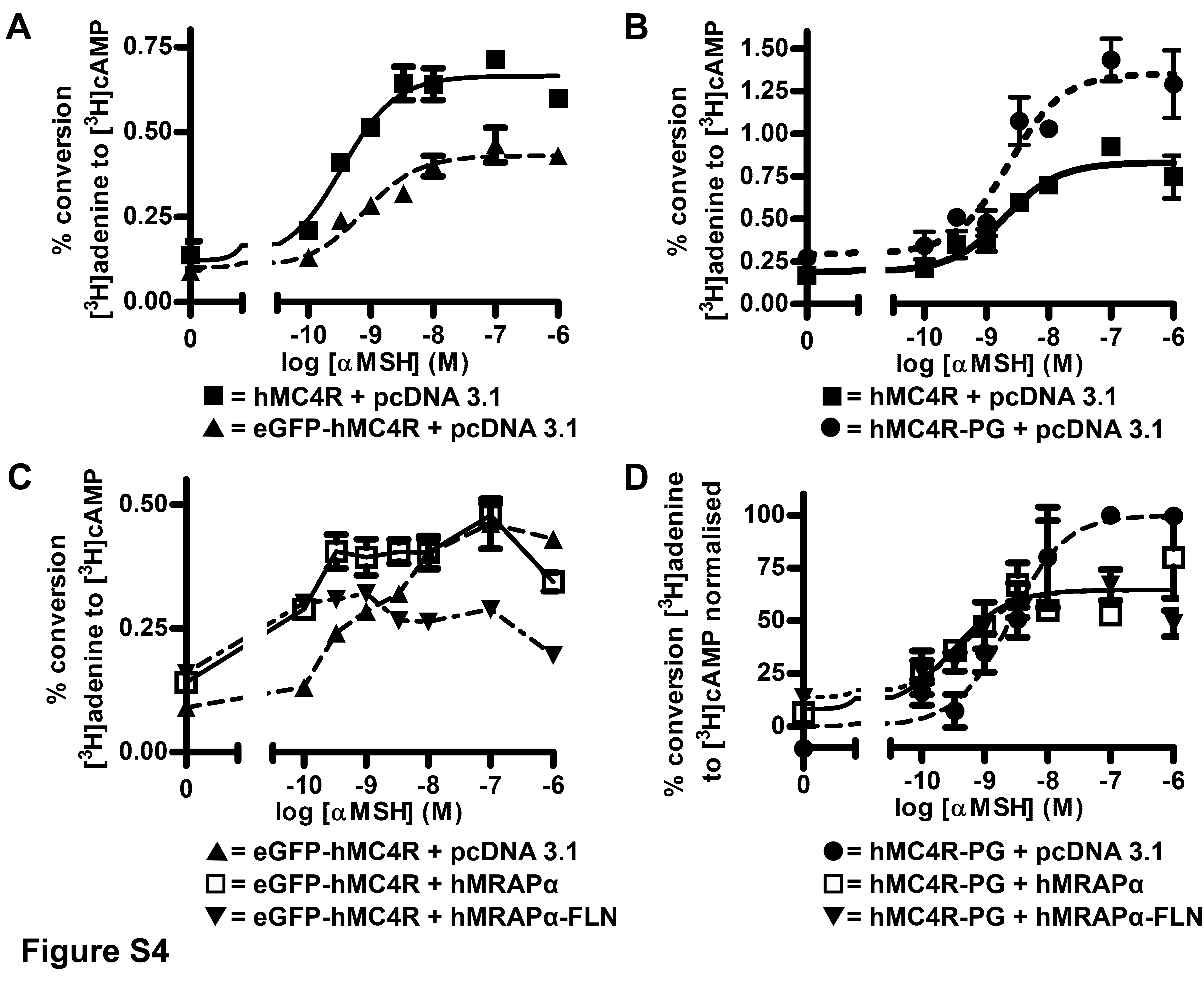

Supplement: S4 Fig — eGFP-hMC4R (A) or hMC4R-PG (B) were transiently transfected in parallel with hMC4R or transiently co-transfected with pcDNA 3.1, hMRAPα or hMRAPα-FLN (C, D). hMC4R, eGFP-hMC4R or hMC4R-PG coupling to adenylyl cyclase was stimulated with increasing concentrations of α-MSH for 1 hour and adenylyl cyclase activity measured. hMC4R, eGFP-hMC4R (A) or hMC4R-PG (B) was transiently co-expressed with the empty vector pcDNA 3.1. hMRAPα or hMRAPα-FLN was transiently co-expressed with eGFP-hMC4R (C) or hMC4R-PG (D). hMC4R, hMC4R-PG and eGFP-hMC4R coupling to adenylyl cyclase were stimulated with increasing concentrations of α-MSH for 1 hour and adenylyl cyclase activity measured. Raw data from 1 experiment performed in duplicate was plotted as mean ± s.e.m. Normalised data from 1 experiment (D) performed in duplicate was plotted as mean ± s.e.m. (TIF) [file pone.0140320.s006.tif]

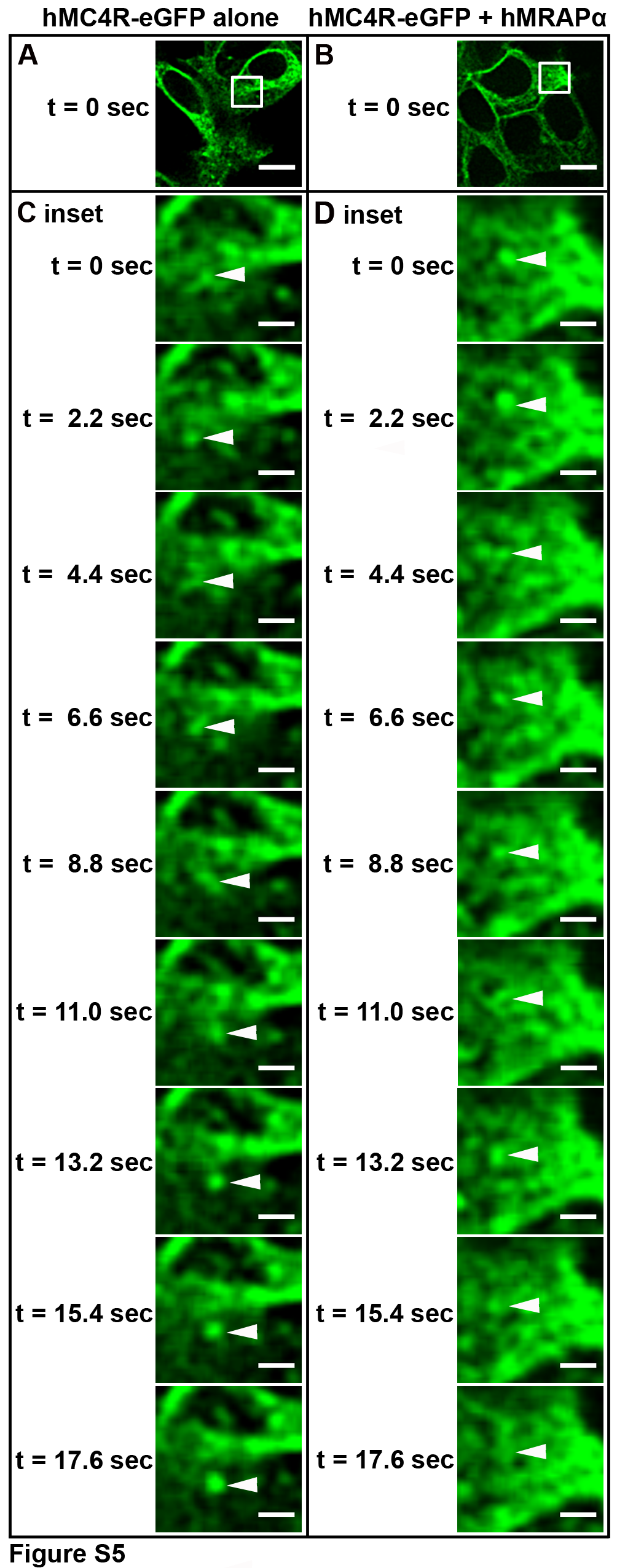

Supplement: S5 Fig — HEK293 cells were transiently transfected with hMC4R-eGFP with pcDNA 3.1 (A, C) or hMRAPα (B, D) and live confocal microscopy was performed with XY scans acquired approximately every 2.2 seconds. Movement of vesicles containing hMC4R-eGFP expression (indicated by white arrows in insets C and D) was observed over time. Scale bars = 10μm, 2μm for insets. (TIF) [file pone.0140320.s007.tif]
